# NaI-Mediated Defluorination: A Mild Route to Reduced Ruddlesden–Popper Oxyfluorides Demonstrated on La_2_CoO_3_F_3_ as Model System

**DOI:** 10.1021/jacs.6c06828

**Published:** 2026-05-25

**Authors:** Jonas Jacobs, Tommi Aalto, Yorik Puffaldt, Clemens Ritter, Oliver Clemens

**Affiliations:** † Faculty of Natural Sciences II, Institute of Chemistry, Inorganic Chemistry, 9176 Martin Luther University Halle-Wittenberg, Kurt-Mothes-Straße 2, 06120 Halle, Germany; ‡ Institute for Materials Science, Department of Chemical Materials Synthesis, 9149 University of Stuttgart, Heisenbergstraße 3, 70569 Stuttgart, Germany; § Institut Laue Langevin, 71 Avenue des Martyrs, 38000 Grenoble, France

## Abstract

We introduce NaI as a mild reagent for low-temperature topochemical defluorination of Ruddlesden–Popper oxyfluorides. In a topochemical fluorination and subsequent defluorination process La_2_CoO_3_F_3_ is obtained from La_2_CoO_4_, using poly­(vinylidene fluoride) (PVDF) or polytetrafluoroethylene (PTFE) as fluorine sources, and converted to La_2_CoO_3_F_2_ by the addition of NaI. Both processes were studied by laboratory *in situ* X-ray diffraction, which reveals a stepwise fluorine uptake through four crystalline intermediates. Subsequent NaI treatment enables controlled F^–^ removal to form La_2_CoO_3_F_2_. This Co­(II) phase is not accessible by direct topochemical fluorination of La_2_CoO_4_ and is not observed along the La_2_CoO_3_F_3_ formation pathway. X-ray and neutron powder diffraction establish La_2_CoO_3_F_3_ as monoclinic (*P*2_1_/*c*) with full occupation of the interstitial anion layer, whereas La_2_CoO_3_F_2_ is isotypic to La_2_NiO_3_F_2_ (*Cccm*) and exhibits a channel-like interstitial anion arrangement. Thermal analysis by *in situ* XRD is used to scan the temperature ranges over which both oxyfluorides retain their structure, and magnetization measurements indicate the change in cobalt oxidation and spin state upon fluorination/defluorination. This study uses the La_2_CoO_4_ → La_2_CoO_3_F_3_ → La_2_CoO_3_F_2_ reaction sequence as model system to demonstrate that, sequential topochemical fluorination and NaI-mediated defluorination provides access to metastable, anion-ordered RP oxyfluorides under milder conditions than conventional hydride-based reductions, avoiding both reduction of the metal cations to their metallic state and anion substitution reactions due to size effects of the iodide ion.

## Graphical abstract

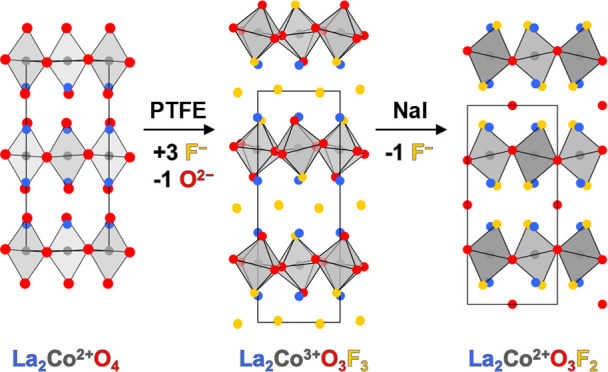

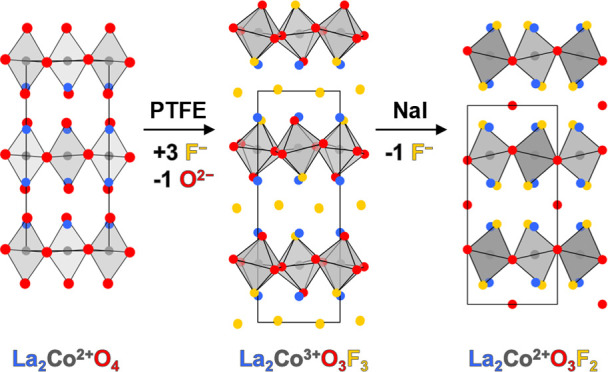

## Introduction

Topochemical modifications of transition metal oxides are a key concept in solid-state material synthesis as they allow the structural modification often at low temperatures making metastable phases accessible, which might not be targeted by direct synthesis approaches. By this, new compounds with improved control over the oxidation states can be obtained targeting compounds with modified physical properties. Oxides of the perovskite related Ruddlesden–Popper­(RP)-type structure (general composition (*A*O)­(*AB*O_3_)_*n*_ with *A,* and *B* being cations) emerged as ideal structural platforms for such topochemical modifications. The RP-type structure consists of *n* layers of perovskite blocks (*AB*O_3_) which are interconnected by *A*O rocksalt-type spacer layers. These spacer layers may serve as a sort of electronic insulation between the perovskite-type blocks leaving RP-compounds as quasi-2D systems. The high structural flexibility regarding cation sizes that is a key feature of the perovskite structure gives rise to numerous compounds with different physical properties and potential applications in catalysis,^1–3^ energy storage,^4–6^ and electronics^7–9^ as well as in fundamental research, e.g., for targeting superconductivity like in La_2–*x*_BaCuO_4+δ_,^10^ or, more recently, La_3_Ni_2_O_7_,^11^ and La_4_Ni_3_O_10_ ^12^ when put under several GPa of pressure. The compositional flexibility in the RP-type structure also expands to the anion content and deviations from the ideal anion stoichiometry are often possible by addition of up to two anions into the AO layer leaving room for additional structural modifications.

Topochemical fluorination of RP-oxides is often used to obtain such anion modified compounds (oxyfluorides) with different physical properties. The oxyfluoride formation can hereby result in three different scenarios:^13^

(1) The reductive replacement of one O^2–^ by one F^–^ anion (*A*_2_ *B*O_3_F).
(2) The redox neutral aliovalent substitution of O^2–^ by two F^–^ anions (*A*_2_ *B*O_3_F_2_).
(3) The oxidative insertion of additional F^–^ anion into the interstitial sites (*A*_2_ *B*O_4_F_*x*_, with *x* ≤ 2).

Additionally, combinations of different scenarios might also be observed. The synthesis of oxyfluorides is often realized by the low-temperature reaction with fluorinated polymers like poly­(vinylidene fluoride) (PVDF) or polytetrafluoroethylene (PTFE) as fluorine sources.^13,14^ These reactions are expected to mainly give scenario 2 and often comprise a highly complex reaction mechanism involving less fluorinated intermediates which can be studied by *in situ* XRD as previously shown for synthesis of the RP-oxyfluorides of the La_2_NiO_4_–La_2_CuO_4_ ^15–17^ substitution series, as well as for the synthesis of La_4_Co_3_O_10_F_2_,^18^ and La_4_Ni_3_O_8.4_F_3.5_.^19^ The reaction with F_2_ gas or AgF/AgF_2_ as fluorine source are further possible and are expected to give scenario 3. Such reactions can also include metastable fluorination intermediates like La_2_CoO_4_F_2–*x*_ as well as La_2_NiO_4_F_2–*x*_ that were previously revealed to exist during the AgF-based fluorination of La_2_CoO_4_, and La_2_NiO_4_ as well as during electrochemical fluorination;^20^ in this study, it was also observed that O_2_ evolution is a key factor at temperatures beyond 250 °C, i.e., keeping the fluorination temperature as low as possible can be considered key in order to stabilize high oxidation states.

In recent investigations, oxyfluorides with less-stable transition-metal oxidation states are obtained by implementing an additional post-synthetic defluorination step yielding, for example, Sr_*n*+1_Ti_*n*_O_3*n*+1–2*x*_F_2*x*–*y*,_ ^21,22^ La_2_NiO_3_F,^23^ Pr_2_NiO_3_F,^24^ LaSr_2_CoRuO_5_F_1.5_,^25^ and La_3_Ni_2_O_5_F,^26^ which are not accessible via direct fluorination. This topochemical defluorination of RP-oxyfluorides is typically realized by reaction with metal hydrides like NaH,^23^ CaH_2_,^24^ and LiH^25^ as reductive defluorination reagents. Such reactions utilize the very high redox potential of the H_2_/2H^–^ couple (*E*_0_ ≈ −2.23 V vs SHE^27^) as well as the formation of stable binary metal fluorides as a driving force. The use of metal hydrides bares the risk of unwanted side reactions like (1) partial F^–^/H^–^ exchange (because of the similar ionic radius) as previously reported for Sr_2_TiO_3_F_2_,^21^ Sr_3_Ti_2_O_5_F_4_,^22^ La_2_NiO_3_F_2_,^23^ and LaBaInO_3_F_2_,^28^ (2) removal of O^2–^ in addition to F^–^ reported for LaSr_2_CoRuO_5.5_F_3.5_,^25^ and (3) the decomposition by further reduction of the metal cation to its metallic state which is why stoichiometry and temperature are very sensible parameters for these reactions. Note that, in the case of RP-oxides, metal hydrides were further successfully used to prepare oxyhydrides by O^2–^/H^–^ exchange like in Sr_2_Mn_0.5_Ir_0.5_O_3.25_H_0.75_,^29^ LaSr_3_NiRuO_4_H_4_,^30^ and La_*x*_Sr_2–*x*_Co_0.5_Ir_0.5_O_4–*y*_H_*y*_.^31^ So far, mainly electrochemical methods have been considered as being highly selective for the defluorination of fluorinated RP-type materials,^6,32–34^ but chemical methods which can avoid anion substitution are lacking at current.

We report NaI as a mild, defluorination agent for a successive fluorination and defluorination strategy in Ruddlesden–Popper oxyfluorides. The fluorination of La_2_CoO_4_ yields La_2_CoO_3_F_3_ (Co^3+^) and NaI enables the controlled conversion to La_2_CoO_3_F_2_ (Co^2+^) while suppressing overreduction to metallic Co and thus preserving the layered framework. This study is centered around the structure, crystal chemistry and magnetic properties of both oxyfluorides while the formation as well as the decomposition reaction are further investigated by *in situ* X-ray diffraction.

## Experimental Section

### Synthesis

The precursor oxide La_2_CoO_4_ was obtained by a citric acid-assisted combustion method similar to that previously described.^20^ Stoichiometric amounts (targeting 5 g final oxide) of La_2_O_3_ (ChemPUR, 99.99%; dried at 900 °C for 10 h), and Co­(NO_3_)_3_·6H_2_O (Sigma–Aldrich, 98%) were dissolved in ∼25 mL of distilled water with the addition of a few drops of concentrated HNO_3_. Citric acid (Anhydrous, Grüssing, 99%; molar ratio metal ions:citric acid = 1:3) was added while stirring. The resulting clear solution was subsequently dried on a hot plate at 120 °C until gel formation. The gel was further heated at 350 °C until ignition. The resulting powders were heated in air at 650 °C for 10 h to ensure complete removal of the organic matrix. The precursor was then calcined at 1300 °C for 12 h in an atmosphere of flowing argon (150 mL min^–1^) yielding La_2_CoO_4_.

The oxyfluoride La_2_CoO_3_F_3_ was synthesized by hand mixing the precursor oxide with either PVDF (Alfa Aesar) or PTFE in a 1:1.5 oxide:polymer ratio using an agate mortar (the monomeric mass of 64 g mol^–1^ (CH_2_CF_2_; PVDF), and 50 g mol^–1^ (0.5 C_2_F_4_; PTFE) were used). The mixtures were slowly (1 °C min^–1^) heated in air to 330 °C (PVDF)/370 °C (PTFE), kept at this temperature for 60 h (PVDF)/18 h (PTFE), and afterward allowed to cool to room temperature in the box furnace.

Topochemical defluorination was performed by dry mixing the oxyfluoride with NaI (Merck, >99.5%, dried at 180 °C before use) in a 1:1 molar ratio using an agate mortar. The mixture was then placed in a tube furnace and subjected to two heating periods of 20 h at 340 °C in flowing N_2_ (100 mL min^–1^) with regrinding.

### Characterization

X-ray diffraction (XRD) patterns were recorded on two different diffractometers: (1) A Bruker AXS D2 Phaser Bragg–Brentano diffractometer using Cu Kα_1,2_ radiation and a LYNXEYE XE-T 1D silicon strip detector (scans were performed in the angular range of 2θ = 8°–110° with a data point resolution of 0.014° and 2 s detection time per data point); and (2) A STOE STADI MP diffractometer equipped with a MYTHEN2 1K (DECTRIS) silicon strip detector (native data point resolution of 0.015°). Scans were performed in transmission geometry with monochromatic Mo Kα_1_ radiation. Patterns used for structural refinements were averaged from three individual scans in the angular range of 2θ = 5°–75° and 90 min total acquisition time per scan.

High-temperature XRD data were also obtained on the STADI MP diffractometer equipped with the HT1 capillary furnace (STOE). For *in situ* XRD scans, samples were filled in capillaries with 0.5 mm external diameter. The capillaries were open to air allowing for an exchange of the reaction atmosphere. Different temperature programs were used consisting of heating and isothermal segments with repeatedly pattern acquisition. An example template for the fluorination with PTFE consist of stepwise (50 °C steps) heating to 370 °C followed by a 24-h hold. After each heating step a diffraction pattern is obtained and during the 24 h hold diffraction patterns were recorded repeatedly (2θ = 8°–38°, 9 min). Sample spinning was employed during the experiments to optimize crystallite statistics.

Neutron powder diffraction data of La_2_CoO_3_F_3_ were recorded at the high-resolution powder diffractometer D2B located at the Institut Laue-Langevin (ILL) in Grenoble, France. Beamtime was granted for proposal 5-23-769.^35^ Measurements were performed for a ∼1.5 g sample (6 mm V-cylinder) at room temperature with λ = 1.594 Å and an acquisition time of 3 h.

Rietveld refinements were performed using the GSAS-II software.^36^ Bond valence sum (BVS) calculations were performed in order to evaluate the anion distribution using the BondStr program implemented in the FullProf Suite.^37^ Here the bond valence sums^38^ and global instability index values (GII) were calculated with the atomic positions derived from the structure refinements. The BVS_*i*_ of a single atom *i* is obtained by

$${\mathrm{BVS}}_{i}=\underset{i}{\sum }s_{i}$$

with *s*_*i*_ as the individual bond valence:

$$s_{i}=\exp\left(\frac{d_{0}-d_{i}}{B_{0}}\right)$$

Here, *d*_*i*_ is the distance of the atom to its neighbors, *d*_0_ is the reference distance, and *B*_0_ is the valence distance constant.

The GII, normalized to the formal valence, is defined as

$$\mathrm{GII}=\frac{\underset{i}{\sum }\left|{\mathrm{BVS}}_{i}-\left|q_{i}\right|\right|\cdot \frac{{\mathrm{mult}}_{i}}{\left|q_{i}\right|}}{N}$$

Here, BVS_*i*_ is the sum over the bond valences around atom *i* as defined above, |*q*_*i*_| is the absolute value of the formal valence of atom *i*, mult_*i*_ is the multiplicity of the atom position, and *N* is the number of atoms in the unit cell. Therefore, GII represents a tool for comparing the deviation of the calculated value from the formal valence, where lower values indicate a more stable anion distribution.

The average oxidation state of Co in La_2_CoO_3_F_3_, and La_2_CoO_3_F_2_ was determined by iodometric titration: ∼20 mg of sample powder was dry mixed with an excess of KI and afterward dissolved in 30 mL of argon-saturated 1 M HCl solution in an Ar atmosphere. The amount of released iodine was determined by titration with a standardized Na_2_S_2_O_8_ solution (0.005 M) immediately after HCl addition. A saturated starch solution was added as indicator right before the end point was reached. The titration was repeated three times.

Characterization of the magnetic properties was performed with the ACMS magnetometer option of a Quantum Design Physical Properties Measurement System (PPMS-9). Powder samples (approximately 100 mg) were loaded in gelatin capsules, which were then attached to the end of a plastic straw in order to minimize diamagnetic contributions. The temperature-dependent magnetic moment was measured in the temperature range of 5–350 K in the DC extraction mode in two external fields of 0.1 and 5 T applying zero-field-cooled (ZFC) and field-cooled (FC) conditions. The superconducting magnet was set from 2 T to zero in oscillating mode prior to the scans, to reduce the trapped flux. Field-dependent measurements were performed at 5 and 300 K, and full hysteresis loops were recorded in the range from 5 T to −5 T. Effective paramagnetic moments were obtained by Curie–Weiss fits and are calculated from the obtained Curie constant (*C*_mol_) by the following relations:

$$C_{\mathrm{mol}}\;(m^{3}\;K\;{\mathrm{mol}}^{-1})=\frac{\mu_{0}\cdot N_{A}\cdot \mu_{\mathrm{eff}}^{2}}{3\cdot k_{B}}$$

where μ_0_ = 4π × 10^–7^ kg s^–2^ m A^–2^, *N*_A_ = 6.022 × 10^23^ mol^–1^, and *k*_B_ = 1.3807 × 10^–23^ kg m^2^ s^–2^ K^–1^.

The Bohr magneton (μ_B_) is defined as

$$\mu_{B}=\frac{e\cdot \hbar }{2\cdot m_{e}}\approx 9.274\times 10^{-24}A\;m^{2}$$

The magnetic moment per atom is then calculated as

μeff⁢ (in⁢ μB)=1μB3·kBμ0·NA·Cmol=3·kBμ0·NA·μB2·Cmol≈6.26×105·Cmol≈797.5·Cmol

## Results and Discussion

### Fluorination of La_2_CoO_4_ with Fluoropolymers PVDF and PTFE

The fluorination of La_2_CoO_4_ with PVDF and PTFE as the F source was in a first step studied by *in situ* X-ray diffraction in ambient air. PTFE was tested as an additional less commonly used fluorine source compared to PVDF to check for differences in the reaction behavior of both fluorinated polymers. A La_2_CoO_4_ sample obtained from citrate synthesis was used as a precursor oxide. This synthesis approach was found to produce more reactive samples for the related La_2_NiO_4_ and La_2_CuO_4_ oxyfluorides, most probably resulting from smaller particle sizes and thus shorter diffusion pathways.^16,17^

As a result of these *in situ* experiments, it was found that stable oxyfluorides are obtained when reacting the oxide with the fluoropolymers in a 1:1.5 molar ratio (oxide to PVDF or PTFE regarding to CH_2_CF_2_ and CF_2_ as monomeric units). The contour plot of the XRD patterns obtained for the reaction with PTFE is shown in Figure 1(b). This reaction readily takes place in air above 300 °C and single-phase products are obtained after 12 to 24 h at 370 °C, with the exact duration depending on the fluorine source. During the first 6 h of the reaction, diffraction patterns of different formation intermediates are clearly observed and are discussed in detail in the SI. The most important outcome is that the fluorination of La_2_CoO_4_ to La_2_CoO_3_F_3_ proceeds via a multistep topochemical mechanism involving three orthorhombic intermediates (orth#1, orth#2, and orth#3), each characterized by distinct unit cell distortions. The progressive elongation of the *c*-axis in orth#3 in the range of 1 Å compared to orth#2 suggests staged fluorine insertion into every second interlayer, analogous to layer wise fluorination in systems like LaSrMnO_4_F_2_.^39^ This is further supported by the consecutive appearance of orth#3 followed by the final monoclinic La_2_CoO_3_F_3_ phase with its ∼ 2.5 Å increased *c* axis. Quantitative Rietveld refinements show that orth#1 persists throughout the reaction, while orth#2 and orth#3 form and disappear rapidly, indicating they are kinetically limited intermediates. This explains the difficulty in isolating phase-pure intermediates via conventional synthesis. A fourth intermediate with monoclinic unit cell was identified in quenched samples, indicating structural flexibility and the potential existence of a metastable intermediate. These findings highlight the complexity of fluorination in layered oxides and demonstrate the power of *in situ* XRD in unraveling multistep topochemical reactions. The reaction with PVDF progresses the same way and is therefore not discussed further.

**Figure 1:**
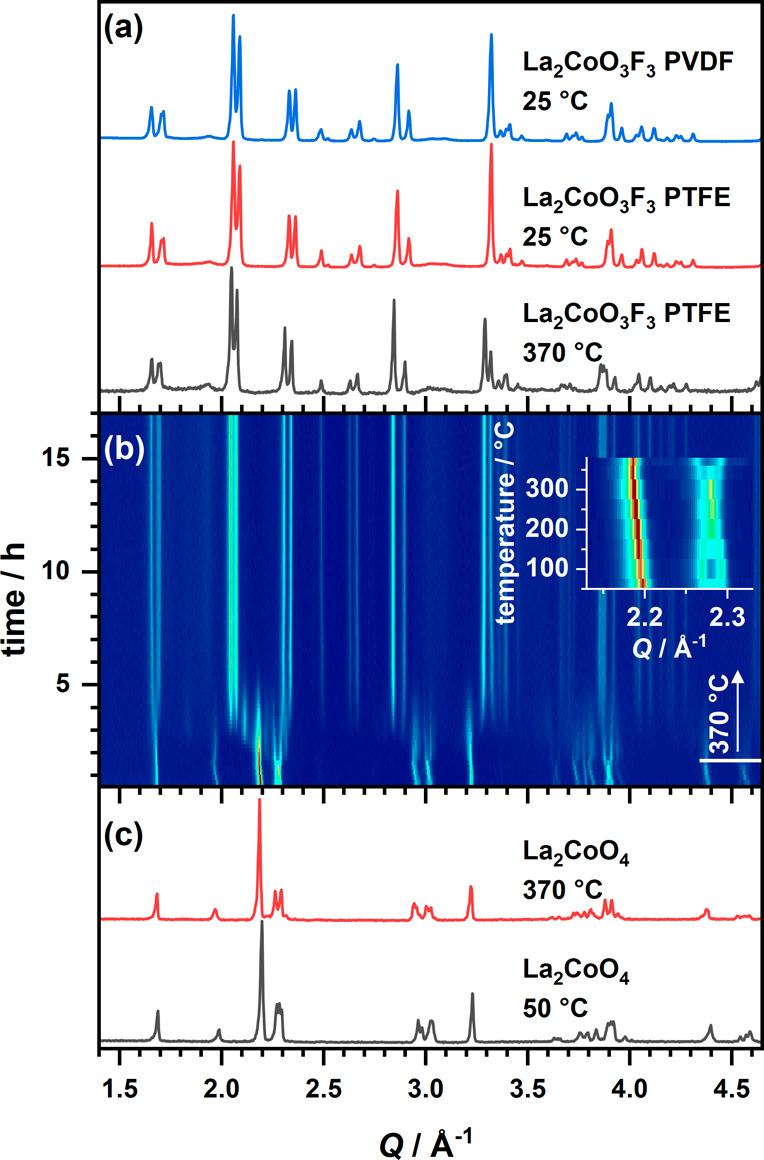
Contour plot obtained for the fluorination reaction of La_2_CoO_4_ with PTFE at 370 °C (b) (heating from 50 to 370 °C was performed during the first 1.5 h and the diffraction patterns for the heating segment are shown for the most intense peak in the inset). The diffraction pattern of the oxide and oxyfluoride are shown in panels (c) and (a), respectively. For the oxyfluoride, the *ex situ* data for samples obtained from PTFE and PVDF as fluorine source are additionally shown.

Bulk synthesis of the oxyfluoride was afterward successfully performed and single-phase products were obtained after 60 h at 330 °C (PVDF) and 18 h at 370 °C (PTFE). The difference in reaction temperature between both compounds results from parameter optimization which was performed due to the different decomposition temperatures of both fluorination reagents. This was required in order to minimize the formation of LaOF/LaF_3_ as decomposition products. A similar behavior was observed for the fluorination of La_2_NiO_4_, where lower reaction temperatures with increased durations were found to produce products with less impurities.^17^

Both fluorination reactions yield powdered samples which appear black to the eye. The diffraction patterns of both samples obtained with PVDF and PTFE are shown in Figure 1(a) and the diffraction patterns of the starting oxide are shown in Figure 1(c). From visual comparison of the diffraction patterns, no significant difference between the products from both fluorination reagents is apparent. When compared to the diffraction patterns of La_2_CoO_4_ a significant shift and clear splitting of the main reflection ((113) at *Q* ≈ 2.1 Å^–1^) can be observed upon fluorination. This points to a monoclinic space group for the oxyfluoride which is consistent with previous results reported on samples from electrochemical fluorination of La_2_CoO_4+δ_ by Nowroozi et al. Here, the authors estimated an overall fluorine content of the obtained samples of La_2_CoO_4_F_*y*_ (*y* ≈ 1.2), according to Faraday’s law.^6^ For this compound, a structure model in space group *C*2/*m* was obtained from XRD for samples from electrochemical fluorination, as well as from attempts to mimic the reaction by using AgF.

For the structural and compositional analysis of the final product La_2_CoO_3_F_3_, neutron powder diffraction data were additionally obtained. This is needed as the structural characterization of oxyfluorides is complicated by the weak X-ray scattering power of the anionic lattice, compared with the large metal cations (La and Co in this case). The almost identical neutron scattering lengths of O and F do not allow for a differentiation between both anions in the NPD data as well, and structure refinements were therefore performed as joint refinements with one XRD and one NPD dataset but without differentiation between the anions (i.e., all anions were first refined as oxygen). By this, the precise anion positions, atomic distances, as well as the total anion content can be obtained from the site occupancy factors (s.o.f.). This information was then used to find the most probable O^2–^ and F^–^ distribution to the anion positions by bond valence sum (BVS) analysis (vide infra).

Refinements were initially attempted in the *C*2/*m* structure model due to the clear similarity to the previously reported compounds.^6^ During these refinements, residual diffracted intensity was clearly present in the NPD data for reflections which are not allowed for the extinction conditions of the used structure model (compare Figure S1). This missing intensity is also found in the XRD data but only when high-intensity data is used. The shown reflections can be indexed as (121), (016), and (122), which demands a primitive unit cell. Final refinements were performed in the highest symmetric primitive monoclinic space group *P*2_1_/*c* with *a* = 5.3058(1) Å, *b* = 5.3741(1) Å *c* = 15.1132(3) Å, β = 91.26(1)°.

The Rietveld plots of the final refinement run are shown in Figure 2, and the obtained crystallographic parameters are listed in Table 1. Fluorination results in a clear elongation of the *c* axis of ∼2.5 Å, compared to the starting oxide La_2_CoO_4+δ_ (*Bmab*, *a* = 5.4740(1) Å, *b* = 5.5345(1) Å, *c* = 12.6385(3) Å). Such strong elongation indicates the insertion of F^–^ into the interstitial sites of the LaO rocksalt-type layer. Similar strong unit cell elongations ranging between 2 Å and 3 Å were also previously found for other oxyfluorides upon fluorination.^6,18,19,39^ In addition to the unit cell elongation in *c*, a contraction of the *ab*-plane is observed, which is accompanied by a clear monoclinic unit cell distortion. This distortion is the result of an increased tilting of the Co­(O,F)_6_ octahedra (compare Figure 3). The observation of such pronounced octahedral tilting together with the strong elongation in *c* is not commonly found for *n* = 1 oxyfluorides in the literature. Usually, tetragonal unit cells with full (or staged) interstitial occupation and strong elongation perpendicular to the perovskite layers but without any octahedral tilt are observed (like in Sr_2_TiO_3_F_2_,^40^ LaSrMnO_4_F,^41^ LaSrMnO_4_F_2_ ^5,34^ ). Alternatively pronounced octahedral tilting with partial occupation of the interstitial sites is found (like in La_2_(Ni, Cu)­O_3_F_2_,^15,42,43^ La_2_(Ni, Cu)­O_2.5_F_3_ ^16,44^ LaBaInO_3_F_2_ ^45^). From refinement of the anion s.o.f. (while constraining *U*_iso_ to be the same for all anion sites), no significant or systematic deviation from full occupation was obtained for all anion sites, which is why these values were fixed to unity in the final refinement run in order to keep the number of refinable parameters reasonable. With robust anion positions obtained from NPD data bond valence sum (BVS) analysis were performed in order to indirectly derive the most probable F^–^/O^2–^ anion distribution to the anion sites. This is done by comparing the resulting global instability index (GII) values, as well as the obtained ion BVS. Here, La and Co were assumed to both adopt the oxidation state +3 which is common for La and which was confirmed by iodometric titration for Co (+2.95(4)). The combination of full occupation of all anion sites together with a total cation charge of +9 results in a O_3_F_3_ stoichiometry for the oxyfluoride. The deviations in anion BVS which was derived from full statistic distribution to all anion sites (global instability index (GII): 17.5%) gave a clear hint to F^–^ occupation of the interstitial sites (X5, and X6) as a BVS of ∼1.5 was found for oxygen on these sites, whereas a BVS of ∼1.0 was found for fluorine on this site. Fluorine was therefore set to occupy the interstitial sites which is in agreement with observations from the literature for example in the elongated but octahedral tilted *n* = 3 oxyfluorides La_4_Co_3_O_10_F_2_,^18^ and La_4_Ni_3_O_8.4_F_3.5_.^19^ For the remaining 1F^–^ f.u.^–1^, three different ordering scenarios were tested (compare Figure S2). These scenarios are:

(1) Statistical distribution of 1F^–^ to the remaining anion sites (GII: 12.2%).
(2) Mixed (50/50) occupation of O^2–^/F^–^ on the apical anion sites (X3 and X4) and occupation of the equatorial sites (X1 and X2) by oxygen (GII: 11.4%).
(3) Ordered occupation of F^–^ on the X3-anion site and O^2–^ on the X4 anion site and occupation of the equatorial sites (X1, and X2) by oxygen (GII: 9.5%).

Since all three scenarios gave similar reasonable cation valences, the GII was used to point out the most possible anion distribution. Here, scenario (3) gave the lowest GII value, and a fully ordered anion distribution of fluorine to the interstitial sites together with alternating O/F occupation of the apical sites seems most likely. The resulting anion distribution is shown in the crystal structure in Figure 3. This structural scenario, with Co^3+^ being located in an octahedron with composition CoO_5_F and shifted away from the center of the polyhedron to the direction opposite to the fluoride ion is very similar to what was found for fluorinated titanates,^21^ which show a similar behavior and local noncentrosymmetry. This can also be rationalized from the symmetry lowering and the splitting of the apical anion site accordingly, allowing for such a shift.

**Figure 2:**
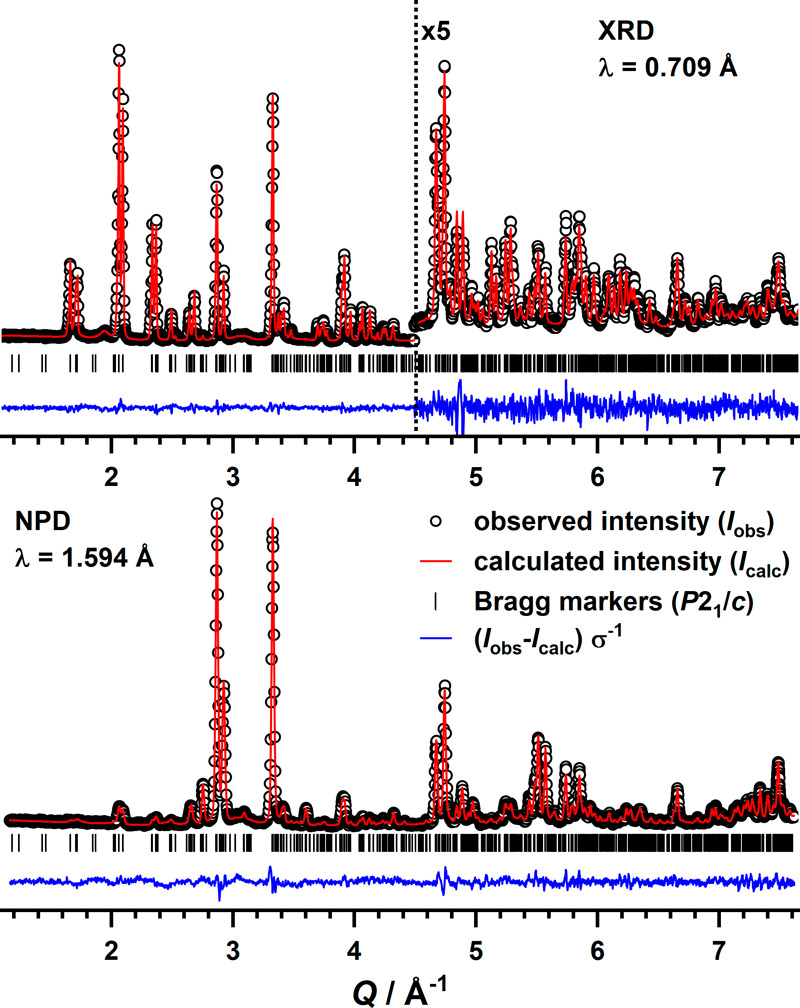
Rietveld plots obtained for the joint refinement of X-ray and neutron powder diffraction data of La_2_CoO_3_F_3_. Refinements were carried out using a structure model with space group *P*2_1_/*c*.

**Table 1:** Structure Parameters Obtained for La_2_CoO_3_F_3_ from Joint Refinements^a^ against XRD and NPD Data

Atom	Anion site	Wyck.	s.o.f.^b^	*x*/*a*	*y*/*b*	*z*/*c*	*U*_iso_ [Å^2^]
La1		4*e*	1	0.2354(8)	0.5035(1)	0.3855(3)	0.0062(7)
La2		4*e*	1	0.2570(8)	0.5021(1)	0.1118(3)	0.0137(1)
Co1		4*e*	1	0.2468(2)	0.0077(2)	0.2531(7)	0.0032(8)
O1	X1/*eq*	4*e*	1	0.5376(1)	0.2030(2)	0.2269(6)	0.0092(2)
O2	X2/*eq*	4*e*	1	0.0482(2)	0.2920(2)	0.2400(6)	0.0080(2)
F1	X3/*ap*	4*e*	1	0.2431(2)	0.5423(2)	0.6280(7)	0.0034(2)
O3	X4/*ap*	4*e*	1	0.2646(2)	0.0544(2)	0.3717(8)	0.0161(2)
F2	X5/*int*	4*e*	1	0.0072(2)	0.7452(2)	0.4911(7)	0.0100(3)
F3	X6/*int*	4*e*	1	0.4998(2)	0.2424(2)	0.0045(8)	0.0011(2)

a Space group: *P*2_1_/*c* *R*_w_ = 5.31%, χ^2^ (NPD + XRD) = 1.16, GOF = 1.08 *a* = 5.3058(1) Å, *b* = 5.3741(1) Å, *c* = 15.1132(3) Å, α = γ = 90°, β = 91.26(1)°, *V* = 430.84(1) Å^3^.

b s.o.f. = site occupancy factor.

**Figure 3:**
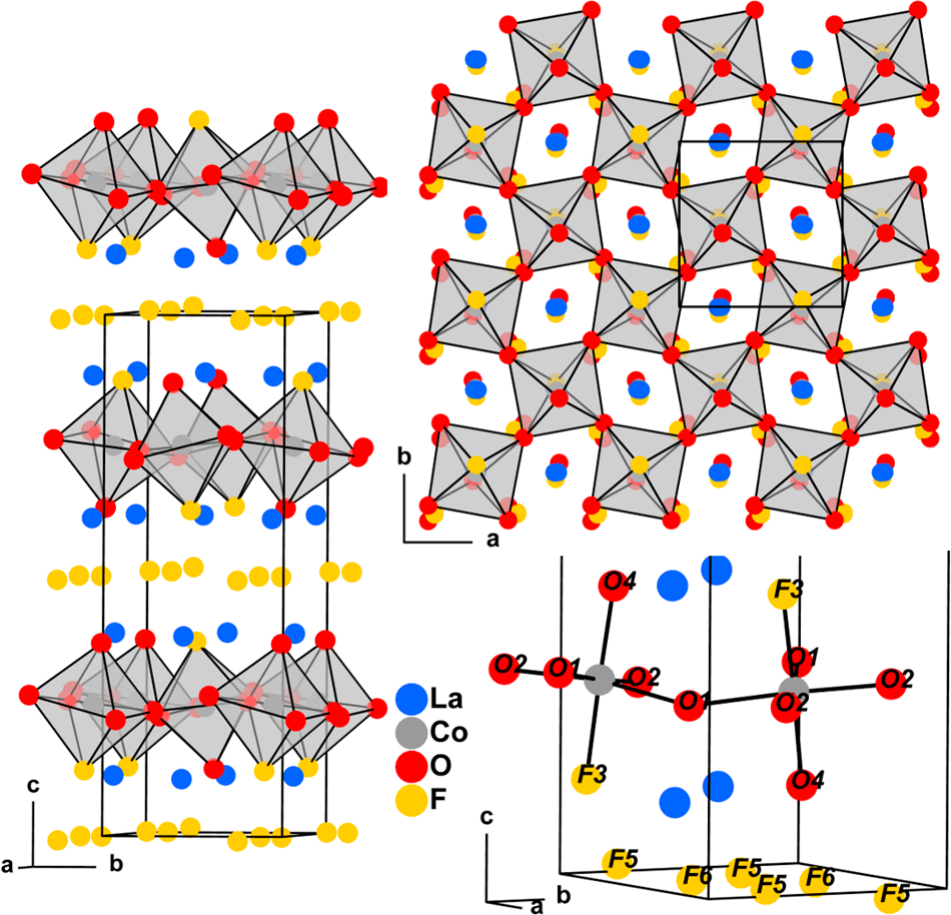
Crystal structure of La_2_CoO_3_F_3_ (space group *P*2_1_/*c*) shown in different viewing directions (please note the individual coordinate system axes). The representation of the Co-coordination polyhedral is also shown.

When comparing the structure and unit cell dimensions of the oxyfluorides of this work with the data for La_2_CoO_4_ oxyfluorides that were previously obtained from electrochemical fluorination and the reaction with AgF differences become apparent. The most important difference is found in the anion stoichiometry of the compounds. Nowroozi et al. derived a La_2_CoO_4_F_1.2_ stoichiometry,^6^ as discussed before, from the amount of electrons used for electrochemical fluorination of this compound and the fact that the fluorination reaction is expected to be oxidative. This stoichiometry would translate to the partial presence of cobalt in the uncommon oxidation state of +4. In our more recent experiments on the fluorination of La_2_CoO_4_ with AgF which were performed *in situ*, we found that a 1:2 (Oxide:AgF) mixture only results in the formation of ∼70% of the oxyfluoride,^20^ while the reaction in a 1:3 ratio results in almost complete conversion to the oxyfluoride. Additionally, we observed the release of oxygen during the fluorination reaction by mass spectroscopy, which is a clear sign that a La_2_CoO_4_F_1.2_ stoichiometry for the AgF product is not plausible. This observation together with the rather similar unit cell parameters (shown in Table 2) facilitates the assumption that the products of the AgF fluorination and the PVDF/PTFE fluorination possess the same structure and similar anion stoichiometry. Small deviations in the unit cell parameters are most likely due to different amounts of defects in the anion lattice and a clear change in the lattice parameters was observed for all fluorinated products after reaching 100% phase fraction during the *in situ* experiments.

**Table 2:** Unit Cell Dimensions for La_2_CoO_3_F_3_ Obtained from Reaction with PTFE, and PVDF as Well as La_2_CoO_4_F_1.2_ from the Reaction with AgF and La_2_CoO_4_F_1.2_ from Electrochemical Fluorination

parameter	PVDF	PTFE	AgF^a^	electrochemical^a^
*a*/Å	5.303	5.309	5.313	5.287
*b*/Å	5.371	5.378	5.382	5.354
*c*/Å	15.169	15.114	15.087	15.292
β/°	91.26	91.24	91.84	91.09
*V*/Å^3^	431.99	431.41	431.31	433.26

a Data taken from ref 6.

The unit cell dimensions of the product that was obtained electrochemically are, on the other hand, significantly different. Here, a more than 0.1 Å increase in the *c* lattice parameter is obtained, together with decreases in the *a* and *b* parameters. This might result from an increased oxygen content and a partial increase of the Co oxidation state above Co^3+^, which is most likely facilitated by the low temperature of 170 °C that was used in the study.^6^ We also emphasize that, in the meantime, a deeper understanding of interdiffusion processes in electrochemical cells with the electrolyte was achieved; thus, interdiffusion processes with the excess of LaF_3_-based electrolyte according to LaF_3_ + La_2_CoO_4_F_1.2_ → La_2_CoO_4−δ_F_1.2+2δ_ + LaF_3–2δ_O_δ_ might also explain why the monoclinic phase can still be observed during electrochemical fluorination,^6^ regardless of only achieving oxidation to the trivalent state of Co. Note, at this point, that the structure of the PTFE compound is the same as for the PVDF compound. Both fluorinated polymers are therefore equally well-suited to obtain La_2_CoO_3_F_3_ and all further data are discussed for a PTFE fluorinated sample.

### Mild Topochemical Defluorination of La_2_CoO_3_F_3_ with NaI

The mild topochemical defluorination of La_2_CoO_3_F_3_ was tested by using La_2_CoO_3_F_3_/NaI in two different ratios: 1:1 and 1:2. Reaction parameters were screened starting from the 1:2 mixture by two different *in situ* XRD experiments, both performed in capillaries, which were open to air. The first experiment consisted of consecutive isothermal plateaus (at least 2 h each) at selected temperatures between 150 and 310 °C. Here, the very slow formation of a different RP-phase was found to start at 310 °C. In the second experiment, the reaction was therefore monitored isothermally at an even slightly increased temperature of 330 °C and high amounts of a new RP-phase were obtained over the course of 19 h (the *in situ* XRD data is shown in Figure 4). The observation of iodine crystals growing at the head of the sample capillary (which is at 20 °C) was seen as first confirmation for the defluorination to happen, according to the reaction

$${\mathrm{La}}_{2}{\mathrm{CoO}}_{3}F_{3}+2x\mathrm{NaI}\to 2x\mathrm{NaF}+xI_{2}+{\mathrm{La}}_{2}{\mathrm{CoO}}_{3}F_{3-2x}$$

Unfortunately, no complete transformation of the O_3_F_3_ oxyfluoride was observed in the *in situ* experiments. The incomplete reaction might result from two different effects: (1) the imperfect mixing of the reagents due to the high crystallinity of NaI and (2) the fact that the *in situ* experiments were performed in open capillaries allowing exchange with the atmosphere most probably allowing partial reoxidation of cobalt with oxygen from the atmosphere. To exclude this potential partial reoxidation, bulk synthesis was later performed in a nitrogen atmosphere.

**Figure 4:**
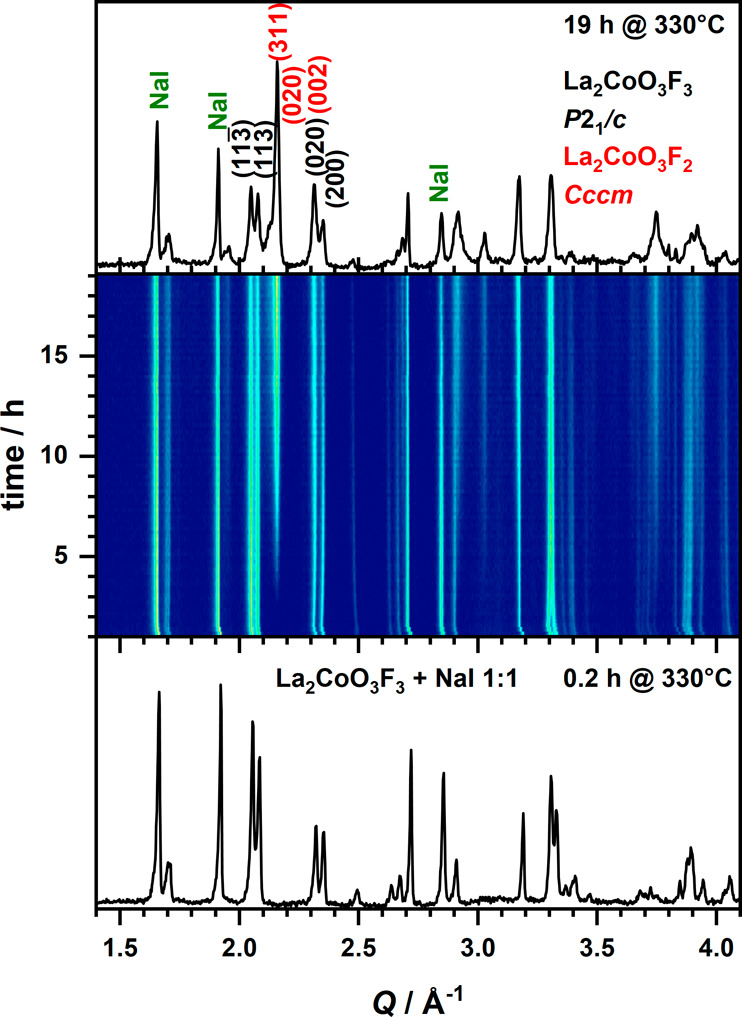
Contour plot obtained for the topochemical defluorination reaction of La_2_CoO_3_F_3_ with NaI (1:1) at 330 °C. The diffraction patterns after reaching 330 °C (bottom panel) and after 19 h (top panel) are additionally shown and the most prominent reflections are indexed.

Almost single-phase bulk samples (referring to the RP phase) were obtained after two reaction steps in flowing N_2_ with regrinding (20 h at 340 °C each). Note that, in experiments with the same reaction mixture, prolonged reaction durations (3 × 20 h at 340 °C without regrinding) did not result in further transformation of La_2_CoO_3_F_3_ to La_2_CoO_3_F_2_. The additional regrinding step is therefore needed due to the high crystallinity of NaI, and the imperfect mixing of La_2_CoO_3_F_3_ is the most probable reason for the incomplete reaction that was observed during *in situ* XRD. Both reaction mixtures (1:1 and 1:2) gave products with similar diffraction patterns consisting of the defluorinated RP-Phase and NaF reflections, while large amounts of unreacted NaI were only found for the 1:2 mixture. The presence of unreacted NaI in the 1:2 mixture was seen as confirmation that NaI indeed only allows the reduction of Co^3+^ to Co^2+^.

The X-ray diffraction pattern of the defluorinated product can successfully be indexed in an orthorhombic unit cell (*Cccm*, *a* = 12.8277(7) Å, *b* = 5.7858(3) Å, *c* = 5.4104(3) Å) similar to the structure of the related Ni-based compound La_2_NiO_3_F_2_. The Rietveld refinement (compare Figure 5) in this structure model gave a plausible fit to the observed intensities. Besides the orthorhombic La_2_CoO_3_F_2_ oxyfluoride phase, NaF, NaI, and a minor impurity with orthorhombic RP-structure (labeled orth#4, refined in *Fmmm*) were included in the refinement.

**Figure 5:**
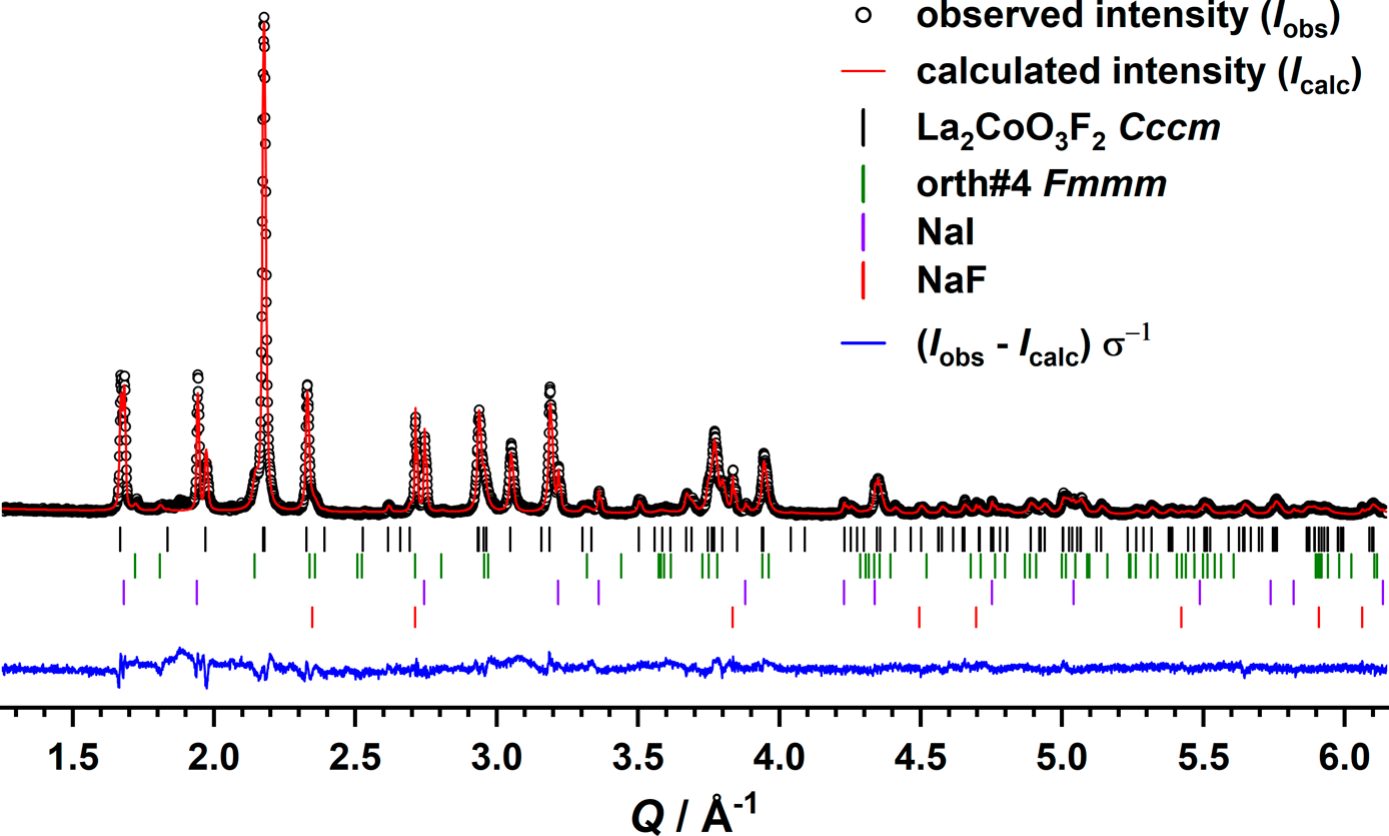
Rietveld plot obtained for the refinement against X-ray powder diffraction data of La_2_CoO_3_F_2_. Refinements were carried out using a structure model with space group *Cccm*. A second orthorhombic RP phase (orth#4), NaF as well as NaI were included as additional phases in the refinement.

The obtained structure parameters are given in Table 3, and the refined crystal structure is shown in Figure 6. The unit cell of La_2_CoO_3_F_2_ has a very strong orthorhombic distortion which is the result of a layerwise octahedral tilting and the formation of a channel-like environment for every second interstitial anion site. This structural distortion was first found and described in detail for La_2_NiO_3_F_2_.^42^ It was also further obtained as monoclinic distorted version for the members of the La_2_Ni_1–*x*_Cu_*x*_O_3_F_2_ substitution series^15,43^ as well as for LaBaInO_3_F_2_.^45^ The orthorhombic strain resulting from the octahedral tilting of La_2_CoO_3_F_2_ with a *b*/*c* ratio of 1.069 is in fact even slightly stronger as for the nickel-containing compound (*a* = 12.8350 Å, *b* = 5.7935 Å, *c* = 5.4864 Å)^42^ with a *b*/*c* ratio of 1.056.

**Table 3:** Structure Parameters^a^ Obtained for La_2_CoO_3_F_2_ from Rietveld Refinements against XRD data

Atom	Anion site	Wyck.	s.o.f.	*x*/*a*	*y*/*b*	*z*/*c*	*U*_iso_ [Å^2^]
La1		8*i*	1	0.3892(1)	0.7400(6)	0	0.0010(7)
Co1		4*e*	1	1/4	^1^/_4_	0	0.0049(8)
O1	X1/*eq*	8*g*	1	0.2714(9)	0	^1^/_4_	0.0263(3)
F1	X2/*ap*	8*l*	1	0.5957(1)	0.6720(2)	0	0.0263(3)
O2	X3/*int*	4*b*	1	0	^1^/_2_	^1^/_4_	0.0263(3)

a Space group: *Cccm*. *R*_w_ = 10.68%, χ^2^ = 2.05, GOF = 1.43 *a* = 12.8277(7) Å, *b* = 5.7858(3) Å, *c* = 5.4104(3) Å, α = β = γ = 90°, *V* = 401.55(4) Å^3^.

**Figure 6:**
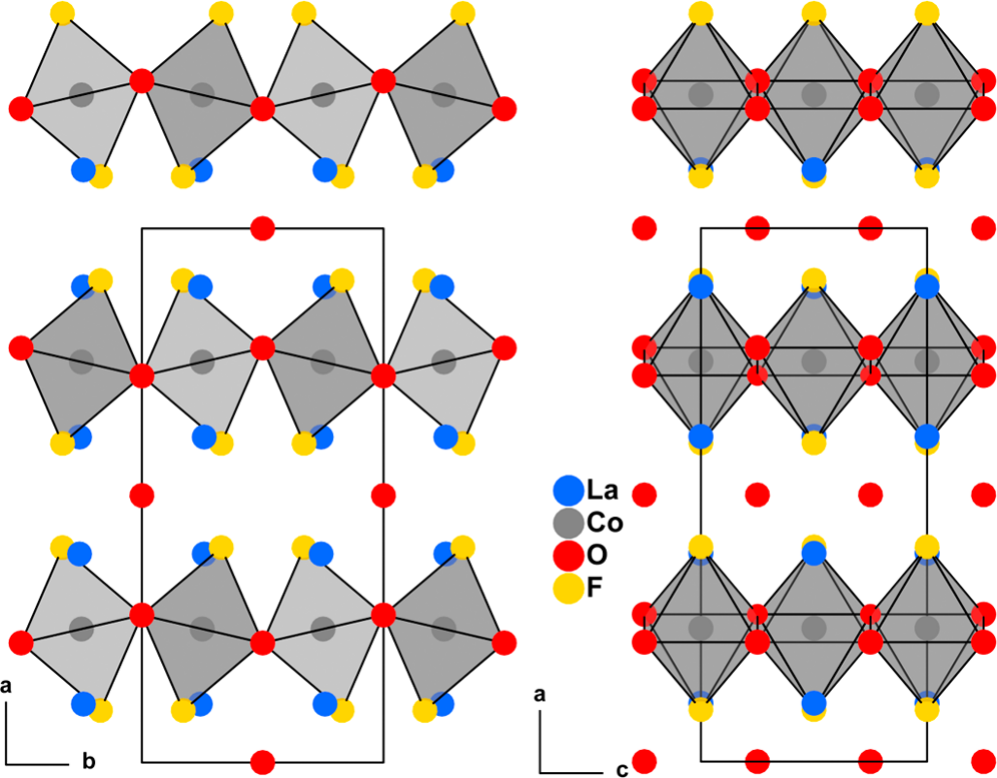
Crystal structure of La_2_CoO_3_F_2_ (space group *Cccm*) obtained from Rietveld refinement against X-ray diffraction data. Two different viewing directions are shown and the anion distribution is given as derived from BVS calculations.

Refining anion positions in the presence of heavy scatterers such as La and Ni is prone to an increased uncertainty in XRD data. Nevertheless, the high symmetry of the *Cccm* structure—featuring only five crystallographic independent atomic sites, three of which correspond to occupied anion positions—still enables a robust refinement of the anion sublattice of La_2_CoO_3_F_2_. To analyze the anion distribution, BVS calculations were performed, assuming La in the +3 oxidation state and Co as Co^2+^, as confirmed by iodometric titration. Four alternative anion distribution scenarios to the three anion sites (octahedral equatorial (*eq*), octahedral apical (*ap*) and interstitial (*int*) were evaluated (compare Figure S10). These four scenarios are described as follows:

(1) Statistic distribution of O^2–^/F^–^ to all three sites (O/F_*eq*_-O/F_*ap*_-O/F_*int*_) (GII: 17.1%).
(2) Pure occupation of the equatorial (*eq*) site with O^2–^ and statistic distribution of O^2–^/F^–^ to the remaining sites (O_*eq*_-O/F_*ap*_-O/F_*int*_) (GII: 13.3%).
(3) Pure O^2–^ at *eq*, pure occupation of the interstitial (*int*) sites by F^–^ and half occupation of the apical (*ap*) sites by O^2–^/F^–^ each (O_*eq*_-O/F_*ap*_-F_*int*_) (GII: 14.0%).
(4) Fully ordered with O^2–^ at *eq*, and *int*, while F^–^ is located on the *ap* site (O_*eq*_-F_*ap*_-O_*int*_) (GII: 11.8%).

From the GII values, the fully anion-ordered model seems to be the most reasonable, and the same O_*eq*_-F_*ap*_-O_*int*_ anion distribution was also found for La_2_NiO_3_F_2_,^42^ highlighting the similarity of both compounds. Interestingly, a similar structure with the same octahedra tilt and anion distribution was recently derived from HR-STEM images as second formation stage for the fluorination of a La_2_CoO_4_ FIB-lamella.^46^ The fact that an intermediate with such structure is not observed during bulk fluorination is most probably due to the limited comparability of the here presented bulk fluorination method with the direct fluorination of a thin film that is prepared as FIB-lamella and underlines the suitability of the NaI defluorination to access reduced bulk phases.

Note that a different ordering scenario of La_2_CoO_3_F_2_ might be expected from the full interlayer occupation by F^–^ and partial F^–^/O^2–^ apical occupation that is found in the parent oxyfluoride La_2_CoO_3_F_3_. The corresponding O_*eq*_-O/F_*ap*_-F_*int*_ scenario nevertheless has a significantly higher GII, which points to an anion rearrangement during defluorination. This is expected to be possible between apical and interstitial anion sites, as high anion mobility is found between these sites in La_2_CoO_4_.^47^ Two possible pathways for such rearrangements, which either start by removing F^–^ from the interstitial sites or the apical octahedral sites, are shown in Figure S11.

The reaction mechanism here provides new opportunities for the combination of topochemical reaction sequences in order to prepare new compounds beyond thermodynamic stability (please note: Investigation of the thermal decomposition behavior are shown in the SI). The 2I^–^/I_2_ redox couple is only moderate (0.536 V vs SHE^27^) and well-suited in order to address redox transitions of 3*d* transition metals. In contrast to hydride-based reductants with H^–^ being similar sized as F^–^/O^2–^, the I^–^ ion is large, and therefore unlikely to be incorporated within RP-type materials due to size effects. Additionally, I_2_ can be removed from the reaction atmosphere by either applying a temperature gradient or using a dynamic atmosphere. This also impacts the redox potential *E* of the reaction by exploiting the entropy term according to Δ*G* = Δ*H* – *T*Δ*S*= −*zFE*, and by this vary the reactivity toward defluorination over a certain range. Similar to using iodides for the lithiation of delithiated cathode materials for lithium-ion batteries,^48^ this reaction route thus provides new capabilities to mimic charging and discharging processes of cathode materials in fluoride ion batteries^49^ to a, so far, unreached extent.

### Magnetic Properties of La_2_CoO_3_F_3_ and La_2_CoO_3_F_2_

The magnetic properties of both oxyfluorides were studied by temperature-dependent susceptibility measurements in the range of 5 to 350 K. These measurements were performed in an applied field of *B* = 0.1 T applying ZFC/FC conditions, as well as at *B* = 5 T during warming. The molar susceptibility (*χ*_mol_) vs temperature data are shown in Figure 7. For the precursor oxide La_2_CoO_4_ antiferromagnetic (AFM) ordering is reported in literature with *T*_N_ strongly depending on the structure and excess oxygen and therefore the amount of Co^3+^ centers in the lattice and being in the order of 30 – 400 K.^50–52^ As the excess oxygen content depends on the history of the sample, regarding synthesis method and storage conditions, we included the *χ*_mol_ vs *T* data of the precursor oxide in Figure 7(a). Both oxyfluorides were synthesized from this precursor to ensure comparability between the samples and their magnetic properties. The La_2_CoO_4_ sample of this work shows nearly temperature-independent behavior in the range between 350 K and 150 K and most probably an AFM transition below ∼35 K, which is seen as a clear peak in the low field data (compare Figure S14) and was also previously reported.^53^ Upon oxidative fluorination and formation of the O_3_F_3_ oxyfluoride a clear change in the magnetic interactions is expected since Co is now in the trivalent oxidation state. Additionally, filling of the interstitial sites might give rise to further exchange pathways between perovskite layers that are separated in the oxide by the NaCl-type spacer layer. The *χ*_mol_ vs *T* data of La_2_CoO_3_F_3_ that is shown in Figure 7(b) clearly resembles Curie–Weiss behavior down to at least 100 K. The inverse susceptibility exhibits a small curvature that must result from a diamagnetic contribution. The data were fitted in the range of 100 to 350 K with an extended Curie–Weiss law 1/χ_mol_ = $\frac{1}{\frac{C_{\mathrm{mol}}}{T-\theta }+\chi_{0}}$ that includes a temperature-independent contribution (*χ*_0_). The fit is also shown as inset and the obtained Curie constant (*C*_mol_) of 4.46 × 10^–6^ m^3^ K mol^–1^ corresponds to a paramagnetic moment of μ_eff_ = 1.7μ_B_ f.u.^–1^. This moment is different then what would be expected for Co^3+^ with *d* ^6^ electron configuration. Here, one would either expect diamagnetic behavior for the low spin (LS) configuration or 4.9μ_B_ for the high spin (HS) case. For LaSrCoO_4_ which also contains pure Co^3+^ a mixing of LS and HS states as well as the occurrence of an intermediate spin (IS) state is reported, which gives rise to comparable effective paramagnetic moments.^54–56^ We therefore assume that this explanation might be also valid for the compound presented here, where a μ_eff_ = 1.7μ_B_ f.u._^–1^_.^–1^value corresponds to ∼65% LS Co^3+^.

**Figure 7:**
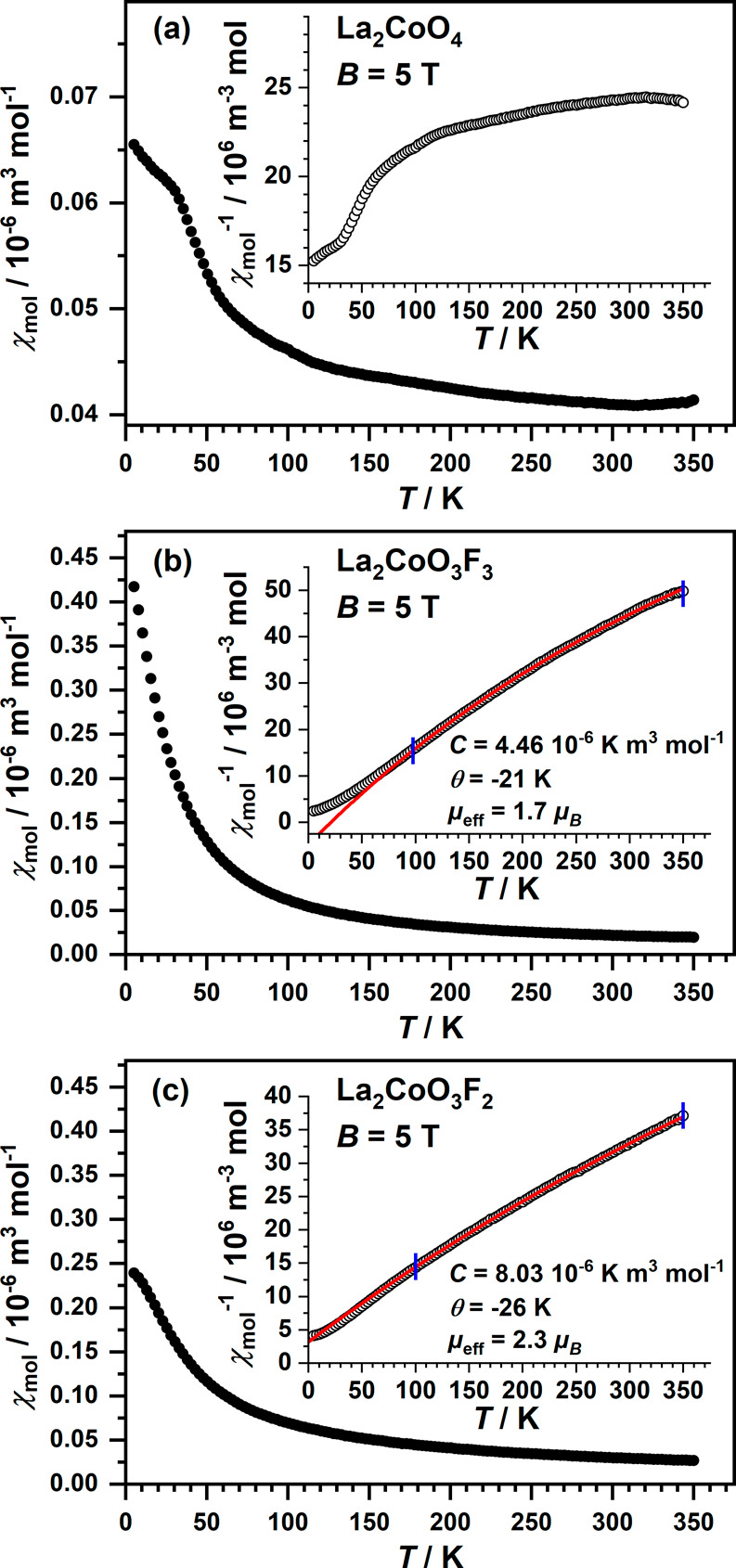
Susceptibility vs temperature data for (a) La_2_CoO_4_, (b) La_2_CoO_3_F_3_, and (c) La_2_CoO_3_F_2_ obtained in a magnetic field of *B* = 5 T. The inverse susceptibility is also shown as an inset including the fit of the Curie–Weiss law to the data.

After reductive defluorination an oxidation state of +2 is expected for cobalt in the sample which translates to a *d* ^7^ electron system for which in oxides a *S* = ^3^/_2_ HS state is expected. The *χ*_mol_ vs *T* scan of La_2_CoO_3_F_2_ that is shown in Figure 7(c) still resembles a Curie–Weiss behavior with small temperature-independent contribution. The obtained *C*_mol_-value of 8.03 × 10^–6^ m^3^ K mol^–1^ is significantly higher and corresponds to a paramagnetic moment of μ_eff_ = 2.3μ_B_ f.u.^–1^. The determination of μ_B_ f.u.^–1^ is subject to a small error as the sample still contains NaF as byproduct. When assuming a molar 1:1 ratio of La_2_CoO_3_F_2_ and NaF, the sample contains about 90 wt % oxyfluoride and the paramagnetic moment would be corrected to about 2.5μ_B_ f.u.^–1^. This value is lower than 3.9 μ_B_ f.u.^–1^ which is expected for Co^2+^ HS from the spin only equation (note that the expected effective moment for Co^2+^ HS might be even higher due to the existence of an unquenched orbital moment). The deviation of the observed effective moment of Co^2+^ from the expected HS value might point to the simultaneous presence of LS Co^2+^ or the formation of an IS state. The presence of Co^2+^ in a LS spin state is not common for oxides as the ligand field splitting of O^2–^ is not significant enough and the same holds for F^–^ as comparably weak ligand. The formation of an IS state is also not reported in the literature, but should, in principle, be possible due to the presence of elongated CoO_4_F_2_ octahedra (*d*Cu–O_*eq*_: 1.999 Å; *d*Cu–F_*ap*_: 2.031 Å). Further investigations are required in order to understand the spin states in more detail, and will be targeted in a follow-up work.

For both oxyfluorides signs of cooperative magnetism are found at low temperatures in the form of a changed curvature of the *χ*_mol_ vs *T* data obtained at 5 *T*, as well as 0.1 *T* (compare Figure S15). For both compounds, a weak ferromagnetic moment is derived from the sigmoidal shape of the field-dependent magnetization measurements obtained at 5 K (compare Figure S16). The sigmoidal shape of the μ vs *B* data might be the result of a canted AFM spin arrangement since Weiss temperatures of θ = −21 K, and −26 K point to AFM interactions for La_2_CoO_3_F_3_, and La_2_CoO_3_F_2_ respectively. The observation of weak ZFC/FC splitting in the 0.1 T data of La_2_CoO_3_F_2_ (compare Figure S15) further points to magnetic frustration which would be consistent with canted AFM. These investigations show that fluorination strongly alters the magnetic properties compared to the parent oxide, and that topochemical defluorination with NaI results in an increase of the paramagnetic moment due to the presence of Co^2+^ without the formation of metallic Co, which would give clear ferromagnetic contribution and that is usually found when using metal hydrides as defluorination reagents.

## Conclusion and Outlook

In this contribution, we applied a fluorination and defluorination strategy to the La_2_CoO_4_ system, providing new conceptual approaches for the synthesis of oxyfluoride materials. We showed that, by applying this approach with PTFE/PVDF as fluorine sources, and NaI as a mild defluorination reagent, two oxyfluorides with distinct structural distortions are obtained as stable products. The direct fluorination product La_2_CoO_3_F_3_ contains Co^3+^ and exhibits a strongly elongated unit cell with fully interlayer occupation. Unlike for other *n* = 1 oxyfluorides, the fluorination and unit cell elongation additionally result in a strong symmetry reduction from tetragonal to monoclinic due to strong octahedral tilting. The formation reaction was studied in detail by *in situ* XRD and three reaction intermediates were identified from this data while a fourth reaction intermediate was found in quenching experiments. Structural information for these less-fluorinated compounds was derived from XRD data for the first time. Here, a staged F^–^ insertion is assumed as mechanism from the presence of an intermediate with ∼1 Å increased longest axis. The defluorinated Co^2+^ compound La_2_CoO_3_F_2_ on the other hand exhibits a strongly strained orthorhombic structure with channel-like interstitial occupation that was previously reported for the related Ni compound La_2_NiO_3_F_2_. The defluorination reaction consists of a single step as seen in the *in situ* XRD experiments that were also used for synthesis parameter optimization. This defluorination step must be accompanied by a structural rearrangement yielding the observed anion distribution. Interestingly, the La_2_CoO_3_F_2_ compound is not found as an intermediate during bulk fluorination and is only accessible via reductive defluorination.

The results of the investigations presented here highlight the use of NaI as a defluorination reagent to obtain less-fluorinated oxyfluorides of compounds with redox active transition-metal cations without risking decomposition from fully reduction of transition-metal cations to the metallic state. We present a conceptional approach to create milder defluorination conditions by using a reductant with higher electrochemical potential as compared to, e.g., sodium hydride. Since the exothermicity of a chemical reaction depends directly on the potential difference of the two reactants in a redox reaction, this helps to keep temperature change during a topochemical reaction low. This must be considered favorable especially for the synthesis of metastable compounds like La_2_CoO_3_F_2_ and La_2_CoO_3_F_3_, which decompose on heating to critical temperatures of ∼100 – 200 °C beyond the actual reaction temperature. In other words, the limited increase of the Fermi level implied by the reaction with NaI is a key to the synthesis of lower-valent oxyfluorides to avoid metal exsolution.^57^ The approach provided in this article can be expected to be applicable to other transition-metal-containing oxyfluorides in future studies, and can provide a pathway for creating novel transition-metal-containing oxyfluorides with interesting physical properties.

## Supplementary Material



## Notes

Comparison of Rietveld fits in *C*2/*m* and *P*2_1_/*c* for the NPD and XRD data of La_2_CoO_3_F_3_. BVS data and selected representations of La_2_CoO_3_F_3_. Additional investigations on the formation of La_2_CoO_3_F_3_. Comparison of XRD patterns of La_2_CoO_4_, and La_2_CoO_4_ + PTFE during heating to 370 °C. Rietveld refinement to the *in situ* XRD pattern obtained during fluorination of La_2_CoO_4_. XRD patterns obtained for products from reactions of La_2_CoO_4_ with PVDF in different ratios. Reaction time dependent evolution of the lattice parameters of La_2_CoO_3_F_3_. Rietveld Plot for the refinement of a XRD diffraction pattern containing La_2_CoO_3_F_3_, and an additional monoclinic compound. BVS data and selected representations of La_2_CoO_3_F_2_. Proposed reaction mechanism for the defluorination of La_2_CoO_3_F_3_. Susceptibility vs Temperature data of La_2_CoO_4_, La_2_CoO_3_F_3_, and La_2_CoO_3_F_2_ obtained at 0.1T. Magnetic moment vs field data for La_2_CoO_3_F_3_, and La_2_CoO_3_F_2_. Additional investigations on the thermal stability of La_2_CoO_3_F_3_, and La_2_CoO_3_F_2_ (PDF)
