# Supplementary material for: NaI-Mediated Defluorination: A Mild Route to Reduced Ruddlesden–Popper Oxyfluorides Demonstrated on La2CoO3F3 as Model System
Source: J Am Chem Soc. 2026 May 25;148(22):23305–15. doi: 10.1021/jacs.6c06828 (PMC13266980; doi:10.1021/jacs.6c06828)
Supplement: Supplementary file 1 [file ja6c06828_si_001.pdf]

NaI-Mediated Defluorination: A Mild Route to Reduced Ruddlesden–Popper Oxyfluorides Demonstrated on $La_2CoO_3F_3$ as Model System

Jonas Jacobs[1], Tommi Aalto[2], Yorik Puffaldt[2], Clemens Ritter[3], and Oliver Clemens[2]*

[1] Martin Luther University Halle-Wittenberg, Faculty of Natural Sciences II, Institute of Chemistry, Inorganic Chemistry, Kurt-Mothes-Straße 2, 06120, Halle, Germany

[2] University of Stuttgart, Institute for Materials Science, Department of Chemical Materials Synthesis, Heisenbergstraße 3, 70569 Stuttgart, Germany

[3] Institut Laue Langevin, 71 Avenue des Martyrs, 38000 Grenoble, France

Email: jonas.jacobs@chemie.uni-halle.de

Supporting Information

Structure evaluation of $La_2CoO_3F_3$

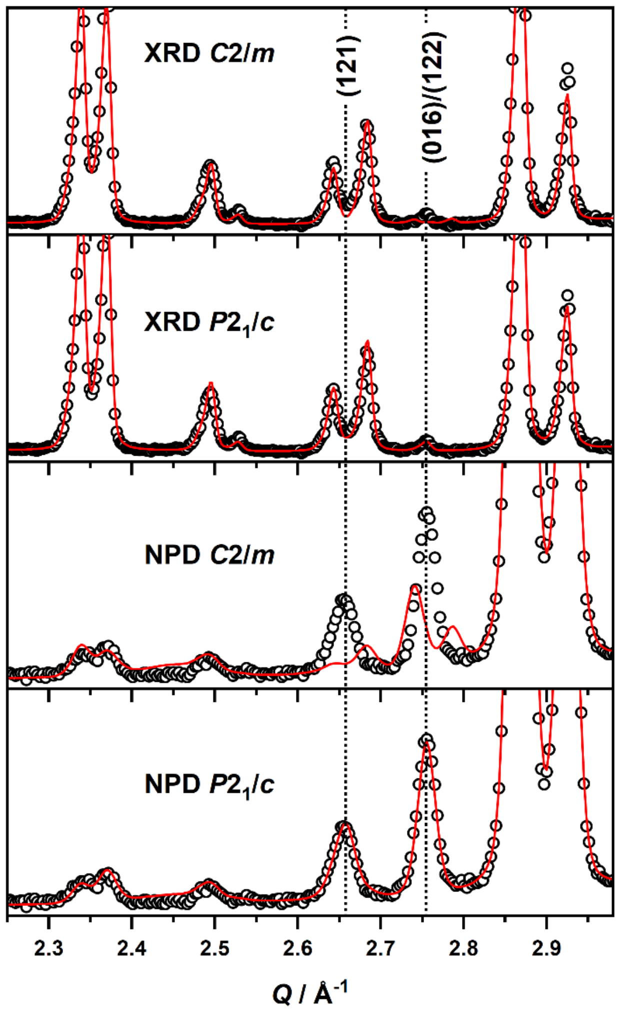

Figure S 1: Observed (circles) and calculated (red line) intensities obtained for refining the structure of $La_2CoO_3F_3$ against XRD and NPD data in two different space groups (*C*2/*m*, and *P*2₁/*c*). The position of peaks with mismatched intensities in *C*2/*m* is highlighted by dotted lines and their (*hkl*) indices are given.

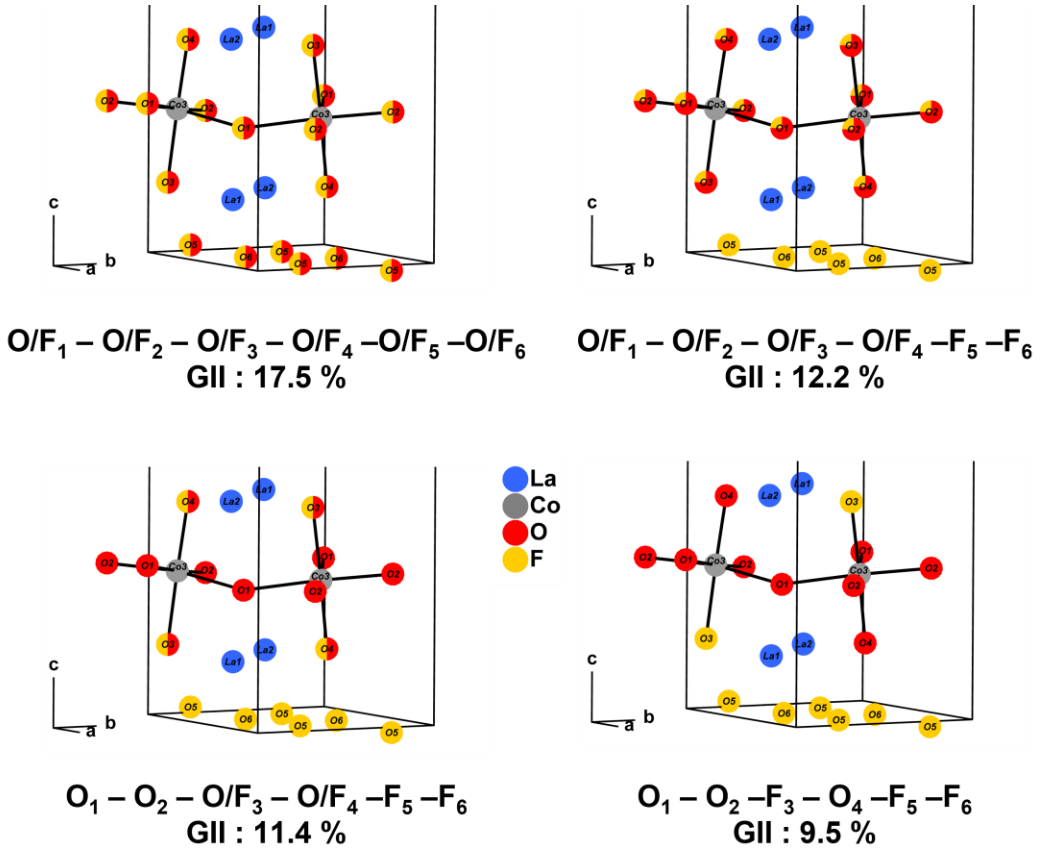

Figure S 2: Partial representation of the unit cell of $La_2CoO_3F_3$ obtained for possible anion distribution scenarios. The global instability index (GII) values obtained from BVS calculations are also given.

The formation intermediates of $La_2CoO_3F_3$

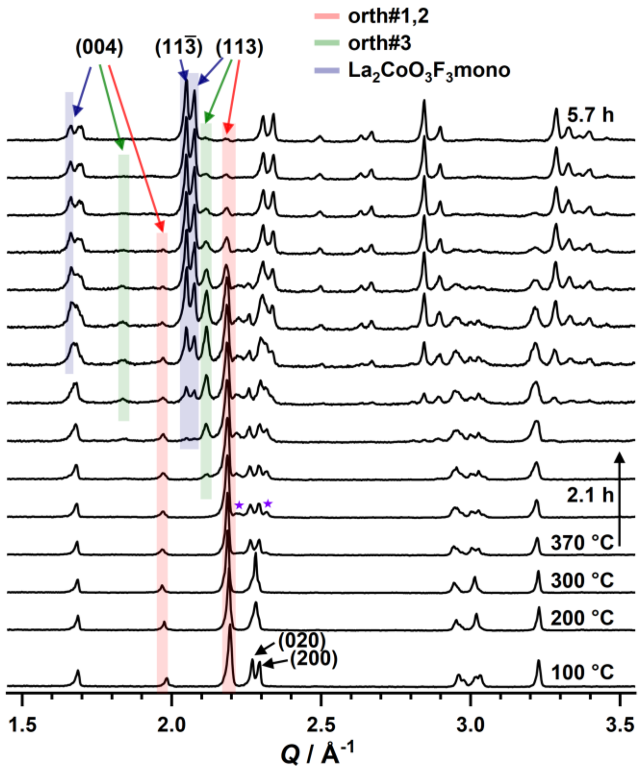

Figure S 3: Selected diffraction patterns obtained during the first 6 h of the fluorination of La_2CoO_4 with PTFE as fluorine source. The position and evolution of the (113) and (004) peaks of two intermediates and the final oxyfluoride are highlighted. Asterisks mark the positions of (020)/(200) of the second orthorhombic intermediate (orth#2).

Like stated in the main text the presence of several reaction intermediates is clearly visible from the in the *in situ* XRD patterns. The staggered occurrence of the individual signals allows them to be assigned to individual reaction intermediates. Diffraction patterns from at least 4 different phases were identified after reaching the reaction temperature of 370°C. In Figure S 3 selected diffraction patterns obtained during the first 6 h of the fluorination experiment show the appearance and disappearance of the different phases. During heating the transition of the unit cell symmetry of the starting oxide from orthorhombic, to tetragonal back to orthorhombic is additionally observed from the merging and subsequent splitting of (020), and (200) between 100 °C and 370 °C. The tetragonal unit cell is linked to the uptake of oxygen by the starting oxide like previously reported[1]. At 370°C the diffraction pattern consists of signals from a first orthorhombic reaction intermediate (denoted orth#1). To check whether this orthorhombic unit cell distortion results from fluorination, the precursor oxide was also heated to 370 °C in air in the absence of a fluorine source. The diffraction patterns of this experiment are shown in comparison to the La_2CoO_4 + PTFE data in the supplement (Figure S 4). The observation of different diffraction patterns above 200 °C in the presence of PTFE was seen as confirmation, that the diffraction pattern after reaching 370°C indeed results from the formation of a first reaction intermediate. A similar

first intermediate was previously observed for the fluorination of La_2NiO_4 which also starts with tetragonal symmetry and the reflections of a first orthorhombic reaction intermediate are already found during heating above 270 °C in presence of PVDF.[2]

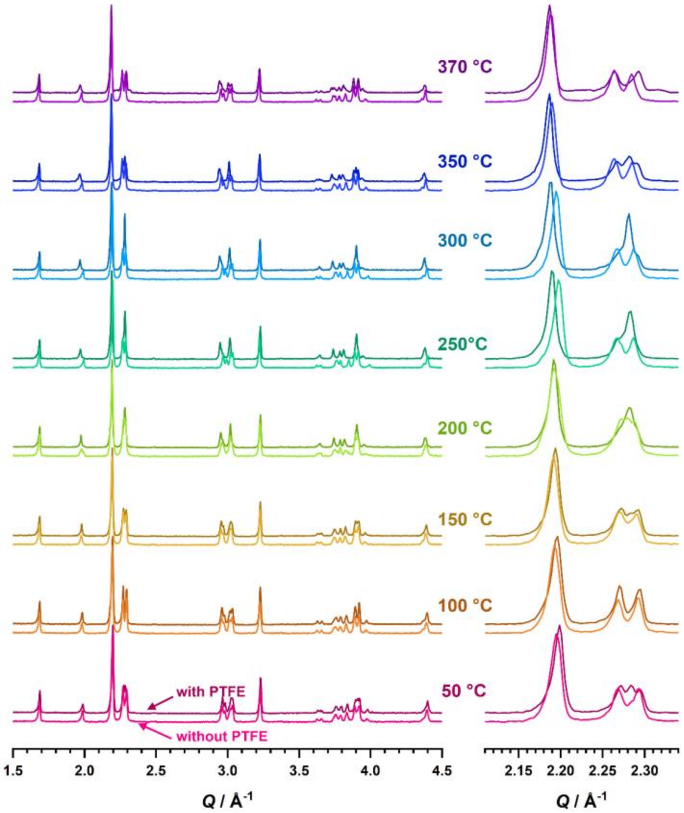

Figure S 4: X-ray diffraction patterns obtained for La_2CoO_4 while heating to 370 °C, either in without (bottom trace) or in presence of PTFE as fluorination source (top trace). The deviation of the diffraction patterns above 200°C points to an onset of the reaction above this temperature. This is why the Phase at 370°C was assumed to be a first reaction intermediate (labelled as ortho#1) instead of assuming pure La_2CoO_4.

In the first scans at 370 °C the emergence of a second orthorhombic phase (denoted orth#2) is observed by the formation of an additional pair of (020)/(200) reflections (indicated with stars in the 2.1 h data of Figure S 3). Here the broadness of the left signal (indexed as (020)) might be interpreted as sign of splitting due to the occurrence of an additional orthorhombic reaction intermediate (like found for the fluorination of La_2NiO_4[2]) but the data is not well enough resolved and is therefore not taken into account. The third intermediate (orth#3) has also an orthorhombic unit cell symmetry but with strongly elongated *c* axis. The elongation in *c* is qualitatively derived from the appearance of the (004) and (113) reflections at significantly lower *Q*-values (highlighted in Figure S 3). As the reaction progresses further the diffraction pattern of $La_2CoO_3F_3$ emerges and the monoclinic unit cell distortion is clearly seen by the splitting of (113) in (11-3)/(113).

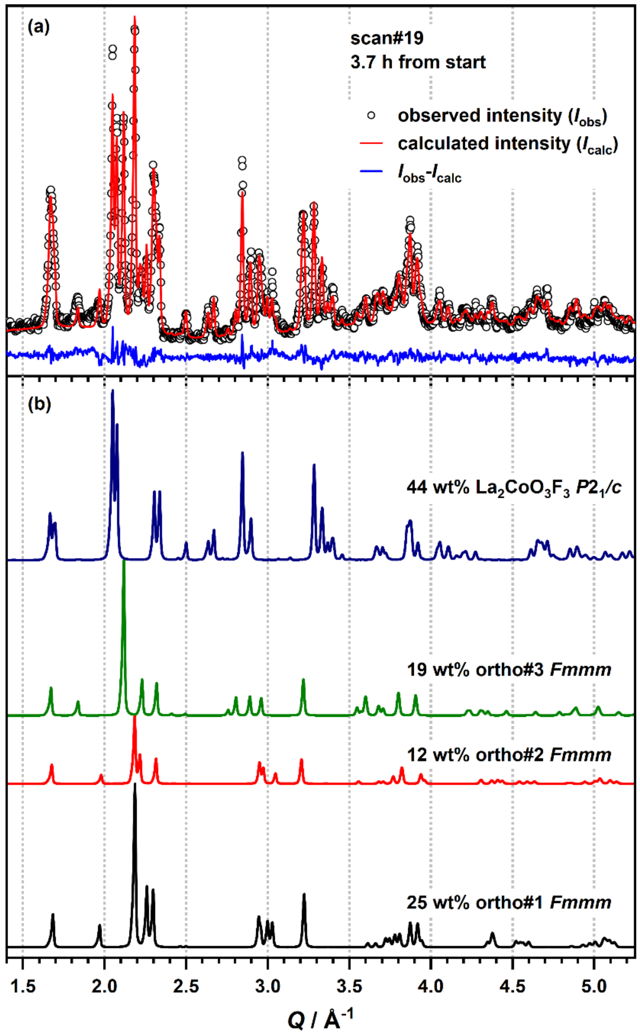

Figure S 5: (a) Observed and calculated diffracted intensity obtained from Rietveld refinement for a selected diffraction pattern (scan#19, 3.7 h in the experiment) from the *in situ* XRD experiment of the fluorination of La_2CoO_4 with PTFE. (b) Simulated diffraction patterns for all 4 observed phases scaled to their refined weight fractions.

To extract the unit cell dimensions as well as weight fractions of the individual reaction intermediates sequential Rietveld refinements were performed for the *in situ* data. The structure parameters of orth#1 and orth#2 were initially extracted from the diffraction pattern recorded after 2.1 h, and were then used in combination with the monoclinic ($P2_1/c$) unit cell of $La_2CoO_3F_3$ to fit the pattern obtained after 3.7 h and to extract the structure parameters of orth#3. This diffraction pattern was chosen as it contains all 4 phases (3 intermediates and $La_2CoO_3F_3$) to a reasonable weight fraction allowing for a robust refinement. A comparison of the observed intensity with the calculated intensity from Rietveld refinement is given in Figure S 5(a) and the simulated diffraction patterns of all 4 phases scaled to their individual weight fractions are shown in Figure S 5(b). The extracted unit cell dimensions as well as weight fractions (wt%) are given in in Table 1. All three orthorhombic intermediates were fitted in space group *Fmmm* being the highest symmetric

subgroup of the tetragonal (*I4/mmm*) K_2NiF_4 aristotype. It has to be noted that for orthorhombic RP-compounds especially with interlayer occupation less symmetric orthorhombic space groups (*Cmca, Cccm, Pbcm*) are often found as they allow for tilting of the CoO_6 octahedra which is not possible in *Fmmm* due to symmetry restrictions. Even though such tilting distortions are expected due to increasing interlayer occupation we nevertheless used the model with *Fmmm* as space group as weak reflections which would indicate different space groups are not well resolved in the present data. For this reason, we will not discuss any changes in atomic positions or *s.o.f*, since they were not refined and only compare the extracted unit cell parameters.

Table 1:Unit Cell Dimensions Obtained for La_2CoO_4 at 25 °C, the Three Orthorhombic Intermediates (orth#1-3) and $La_2CoO_3F_3$ at 370°C after 3.7 h Reaction Duration and $La_2CoO_3F_3$ at 370 °C From the Final Scan (after 22 h) of the *in situ* XRD Experiment. For the Phases Present at 3.7 h the Weight Fractions (wt%) are Displayed.

	**Oxide** 25°C	**orth#1** 370°C 3.7 h	**orth#2** 370°C 3.7 h	**orth#3** 370°C 3.7 h	**$La_2CoO_3F_3$** 370°C 3.7 h	**$La_2CoO_3F_3$** 370°C 22 h
a / Å	5.474	5.462	5.420	5.412	5.369	5.356
b / Å	5.534	5.555	5.660	5.628	5.445	5.434
c / Å	12.638	12.736	12.677	13.655	15.061	15.127
β / °	90	90	90	90	91.08	91.09
V / $Å^3$	382.89	386.49	388.93	415.74	440.25	440.16
wt%	--	25	12	19	44	--

When comparing the unit cell dimensions of the oxide, and the first two intermediates (orth#1 and orth#2) a clear increase of the orthorhombic strain (i.e., the a/b ratio) with progressing reaction is apparent. This might be interpreted to result from an increased octahedral tilting and was previously observed for partially fluorinated compounds during the fluorination as well as defluorination of La_2NiO_4[2] and $La_2NiO_3F_2$[3] respectively. In recent STEM investigations performed for the fluorination products of La_2CoO_4 FIB lamella this was interpreted to result from increasing occupation of the apical CoO_6 octahedra sites by fluorine atoms.[4] For orth#3 the orthorhombic strain is less pronounced and a strong increase of the longest axis is simultaneously observed. This increase, which amounts to almost 1 Å when compared to orth#2, is significantly higher than what is found for $La_2NiO_3F_2$ ($\Delta c \approx 0.2$ Å) as well as for $La_2NiO_{2.5}F_3$ ($\Delta c \approx 0.3$ Å) where fluorination results in similar unit cell volume increase but with strong octahedra tilting instead. The Δc value of ~1 Å is in fact in the range of what is found for staged F-insertion into every second interstitial layer like it is found for $LaSrMnO_4F$[5,6] even though such layer wise fluorine insertion usually gives compounds with tetragonal unit cell. Based on these analogy considerations, we therefore interpret orth#3 as partially fluorinated reaction intermediate containing at least 1 F^- f.u.$^{-1}$ most probably located in every second interstitial layer. The consecutive presence of orth#3, with F^- occupying every second interlayer and $La_2CoO_3F_3$ with full interlayer occupation points to a staged F^- insertion mechanism for the fluorination of La_2CoO_4.

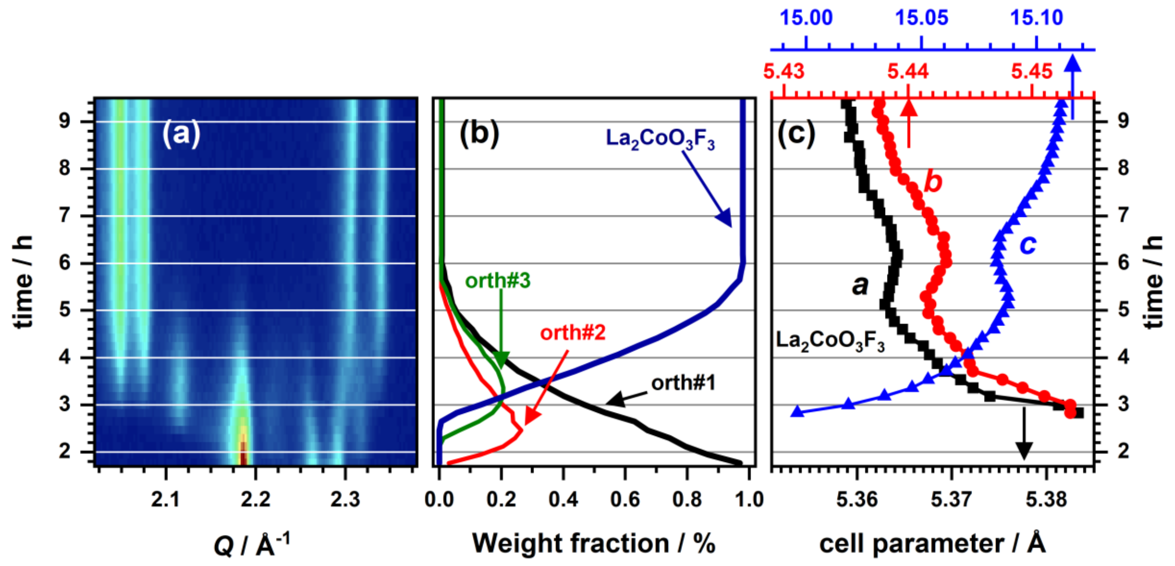

Figure S 6: (a) Selected section of the *in situ* XRD-patterns obtained for the fluorination of La_2CoO_4 with PTFE after reaching 370°C. (b) Weight fraction of the four different phases obtained from sequential Rietveld refinement. (c) Change of the unit cell dimensions (*a*. *b*. and *c*) of the final oxyfluoride $La_2CoO_3F_3$ during fluorination.

From sequential Rietveld refinements to the *in situ* data shown in Figure S 6(a) the time dependent change of the weight fractions of all four phases was extracted and is shown for the first 9.5 h of the reaction in Figure S 6(b). The simultaneous presence of all three orthorhombic intermediates indicates that the formation of the intermediates orth#2 and #3 is the limiting step. This is supported by the fact that the ortho#1 phase exists throughout the whole reaction and only vanishes after 100 wt% of the final monoclinic oxyfluoride phase is reached. By this, the reason is found why isolating phase pure oth#2 and orth#3 by targeted direct synthesis or quenching is not easily achievable as the resulting product will always contain all 3 other phases. And indeed, clear phase mixtures were obtained while attempting targeted synthesis (see Figure S 7). Possibly, this might be related to the previously described observation[7] that the diffusivity of F^- ions within the interstitial sites is also related to the detailed composition of these interlayers, and that O^{2-} on the interstitial sites can also impact and reduce F^- mobility. Thus, the observation that orth#2 and #3 disappear at a faster rate compared to orth#1 might point to such influences impacting the different fluorination kinetics.

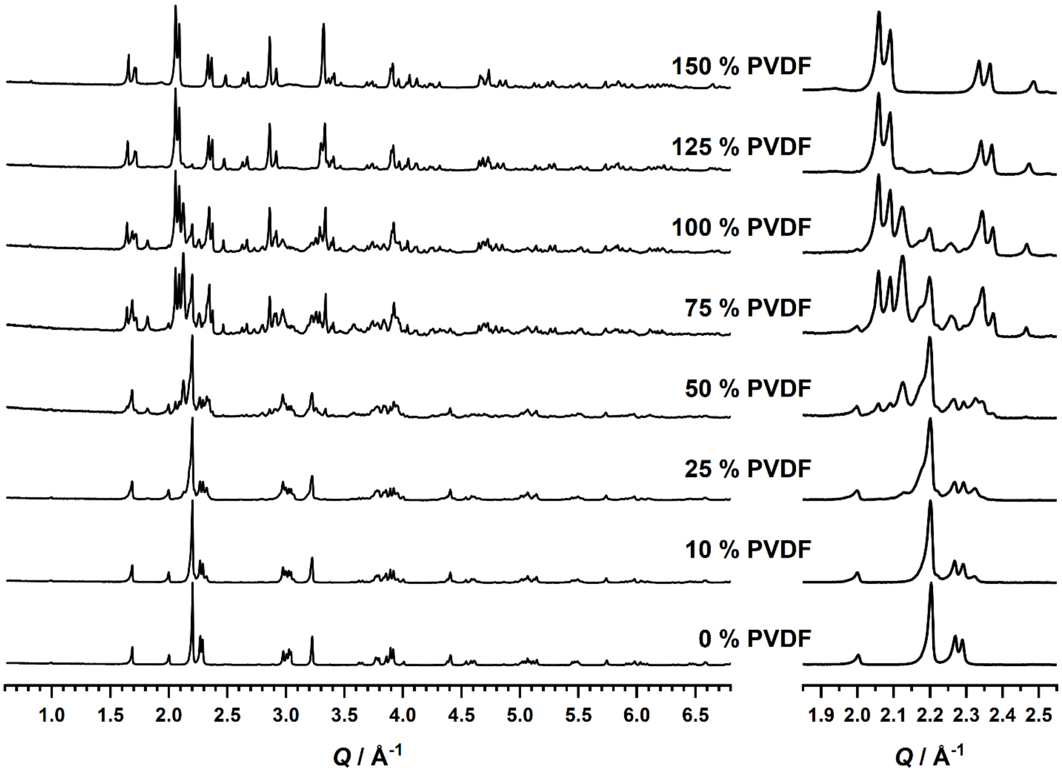

Figure S 7: X-ray diffraction patterns obtained for La_2CoO_4 + PVDF mixtures (0 %, 10 %, 25 %, 50 %, 75 %, 100 %, 125 %, and 150 % PVDF) after they were heated at 330 °C for 60 h. The presence of multiple phases underlines the inaccessibility of the reaction intermediates by this approach.

The time dependent evolution of the lattice parameters, *a*, *b*, and *c* of the monoclinic oxyfluoride phase is additionally shown in Figure S 6(c) (note: the monoclinic angle β is not shown but can be found together with error bars for all parameters in Figure S 8). For the three lattice parameters (*a*, *b*, and *c*) a clear time dependent change is observed. During formation (i.e., while the other intermediates are present) the unit cell of this compound expands perpendicular to the perovskite layers which is seen by a strong increase in *c* of ~0.1 Å that is accompanied by a simultaneous decrease of *a* and *b* (~0.02 Å). This is interpreted to most likely be linked to a filling of the interstitial sites with F^- in the beginning of the oxyfluoride formation. After ~100 wt% of $La_2CoO_3F_3$ is reached (at ~5 h) a deviation from the monotonous unit cell parameter evolution is seen by a clear decrease of *c* accompanied by an increase of *a*, and *b*. This points to changes in the anion lattice even after formation. One possible explanation might be the partial replacement of apical oxygen atoms by fluorine atom leading to the final structure. This interpretation is facilitated by the above discussed fact that electrochemically obtained $La_2CoO_4F_{1.2}$[8] has a longer *c* axis then

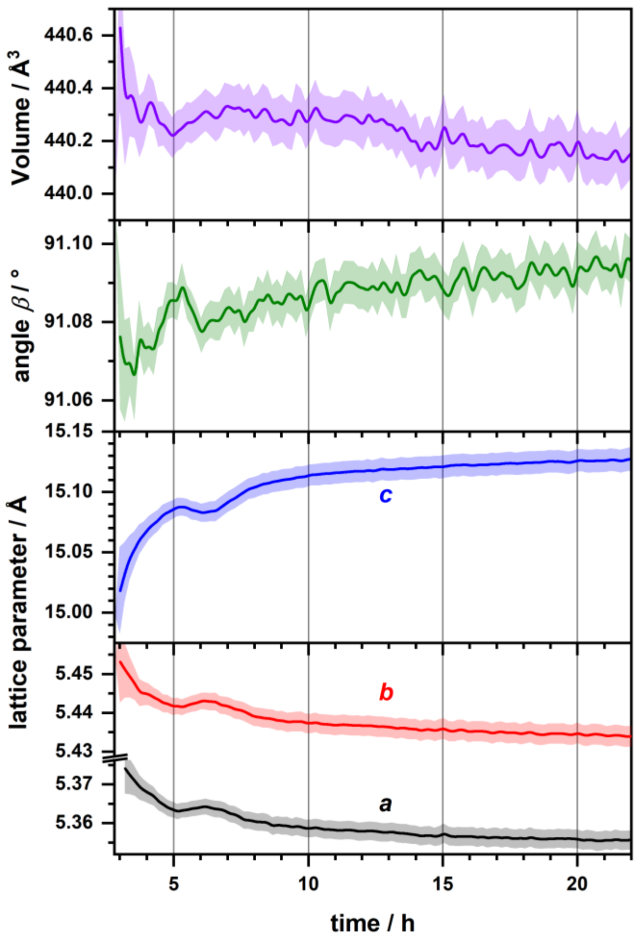

Figure S 8: Reaction time dependent evolution of lattice parameters and volume obtained for $La_2CoO_3F_3$ from the *in situ* XRD experiment.

$La_2CoO_3F_3$. This observed change in the lattice parameter evolution might also point to the presence of an additional formation intermediate with high structural similarity to the final oxyfluoride as reported for the fluorination of La_2NiO_4 yielding $La_2NiO_{2.5}F_3$[2]. The present *in situ* data resolution does not allow for a reasonable refinement with two monoclinic compounds. A larger bulk sample containing two monoclinic phases (1:2 ratio) with significantly different unit cell parameters (especially c, c_1: 15.095 Å, c_2: 15.228 Å) was nevertheless obtained through quenching (the Rietveld plot is shown in Figure S 9). This observation strongly underlines the existence of a further fluorination intermediate with monoclinic structure. After ~6.5 h the change in lattice parameter evolution returns to the initial observed increase of *c* together with a decrease of *a* and *b*. This trend is observed for the full-time scale used, even after 22 h no constant lattice parameter is obtained (compare Figure S 8). Similar observations were previously made for the lattice parameters of the oxyfluorides from the $La_2Ni_{1-x}Cu_xO_3F_2$ substitution series and were interpreted to result from healing of defects in the lattice[9]. From these observations we conclude

that the monoclinic structure of $La_2CoO_3F_3$ most probably possesses a high flexibility of the anion lattice.

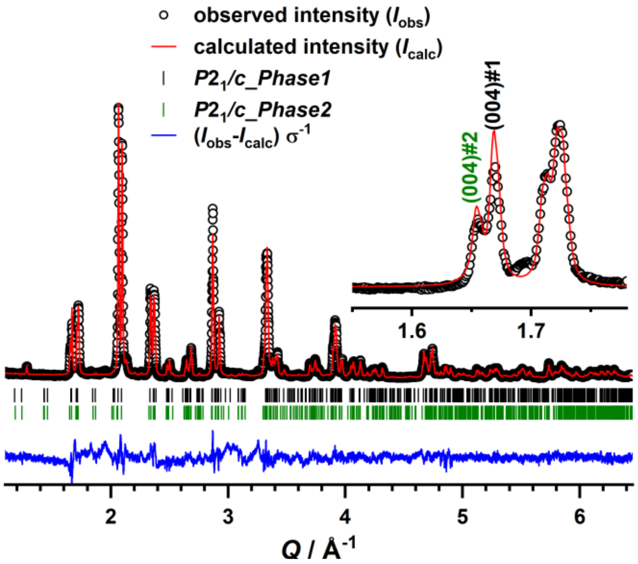

Figure S 9: Rietveld plot from the refinement of a quenched $La_2CoO_3F_3$ sample containing two monoclinic phases with different *c* parameters. In The inset the position of the two (004) Bragg peaks is indicated. (Phase1**:** *a* = 5.316 Å, *b* = 5.384 Å *c* = 15.095 Å, *β* = 91.24°, Phase2: *a* = 5.297 Å, *b* = 5.363Å *c* = 15.228 Å, *β* = 91.26°.

Anion distribution in $La_2CoO_3F_2$

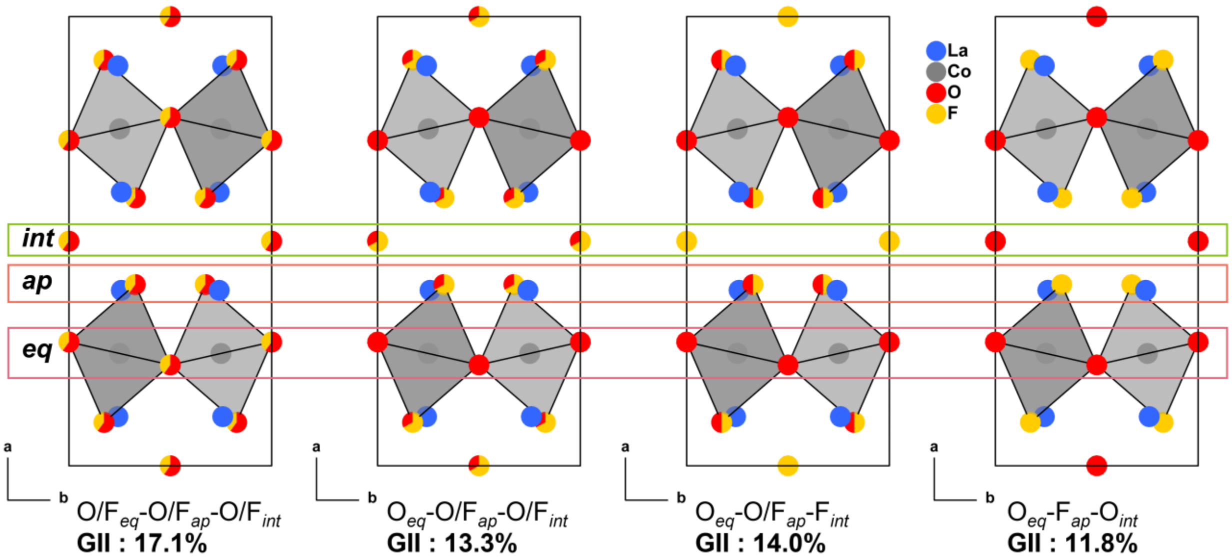

Figure S 10: Representation of the unit cell of $La_2CoO_3F_3$ obtained for possible anion distribution scenarios to the three anion sites labelled as interstitial (int), apical (ap), and equatorial (eq). The global instability index (GII) values obtained from BVS calculations are also given.

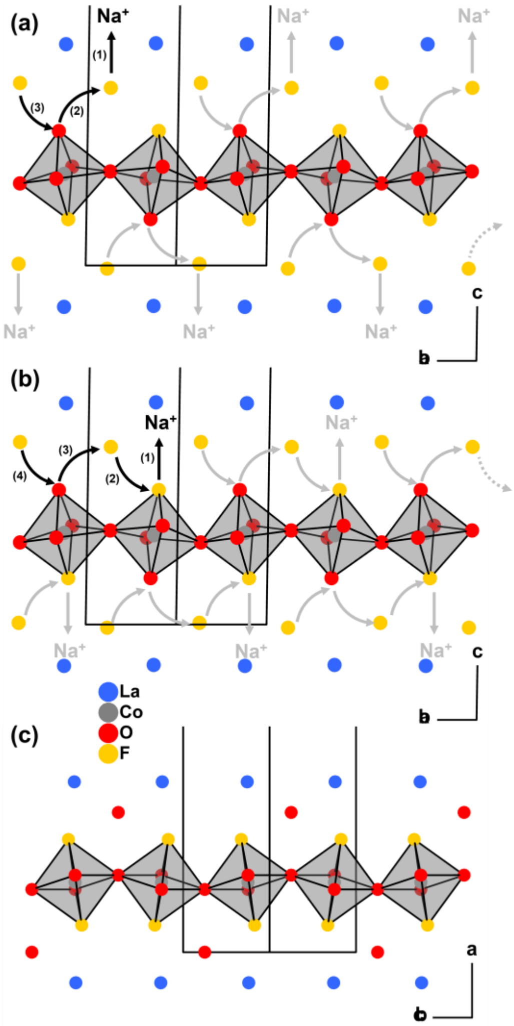

Figure S 11: Selected atoms forming one $Co(OF)_6$ octahedra-strand from the unit cell of $La_2CoO_3F_3$ (a, b) highlighting possible anion rearrangement during reductive defluorination**.** In (a) the rearrangement starts with removing F^- from one interstitial site under formation of NaI and reduction of Co^{3+} to Co^{2+}. The apical O^{2-} ions then migrate to the now unoccupied interstitial site (2) and the remaining interstitial F^- then migrates to the now empty apical sites (3). In (b) the rearrangement starts with removing F^- from its apical position under formation of NaI and reduction of Co^{3+} to Co^{2+}. The now vacant apical site is filled with F^- from one interstitial site (2) which in return is filled by the apical O^{2-} (3). The resulting apical vacancy is then filled by the second interstitial F^-. Both paths leaving the anion distribution observed for $La_2CoO_3F_2$ (c) as result.

Thermal stability of $La_2CoO_3F_3$ and $La_2CoO_3F_2$

The thermal stability and decomposition behavior of both compounds was investigated by *in situ* XRD: Both experiments were performed in open capillaries allowing the exchange with ambient atmosphere. The contour plots from both experiments are shown in Figure S 12 for the temperature range of 350 – 740 °C. For both compounds a clear shift toward lower *Q*-values is evident due to the thermal lattice expansion. For the parent oxyfluoride $La_2CoO_3F_3$ the vanishing of the reflections of the monoclinic unit cell is found at ~520 °C. At this temperature new signals additionally emerge, that can be attributed to a compound with tetragonal RP-type structure. Based on the position of the reflections this decomposition intermediate has increased *a*/*b* axes, and a decreased *c* axis. During formation of this phase signals of LaOF also start to emerge and above ~590 °C the diffraction pattern consists of signals from LaOF, LaF_3, $LaCoO_3$, and Co_3O_4 indicating complete thermal decomposition. For $La_2CoO_3F_2$ thermal decomposition also progresses through the formation of a decomposition intermediate which in this case exhibits the reflection pattern of a RP-compound with less orthorhombic straining and an increased longest axis. This assumption is derived from the shift of the most intense (113) peak to lower *Q*-values. The transition to this decomposition intermediate takes place at ~490 °C and a very similar diffraction pattern was also

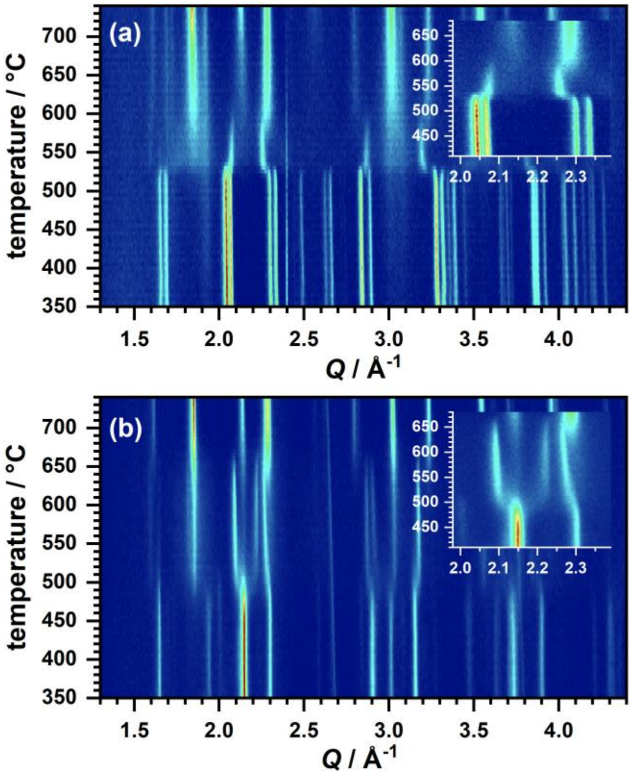

Figure S 12: Contour plots of the temperature depended XRD patterns obtained in the region of 50 to 750 °C for the thermal decomposiiton of (a) $La_2CoO_3F_3$, and (b) $La_2CoO_3F_2$. The enlarged region of the most intense reflections is shown as inset in both plots.

observed during the thermal decomposition of $La_2NiO_3F_2$ with the same transition temperature.[10] This decomposition intermediate has a higher temperature stability then the one observed for the O_3F_3 and the same decomposition products are found above ~650 °C, like for the O_3F_3 oxyfluoride.

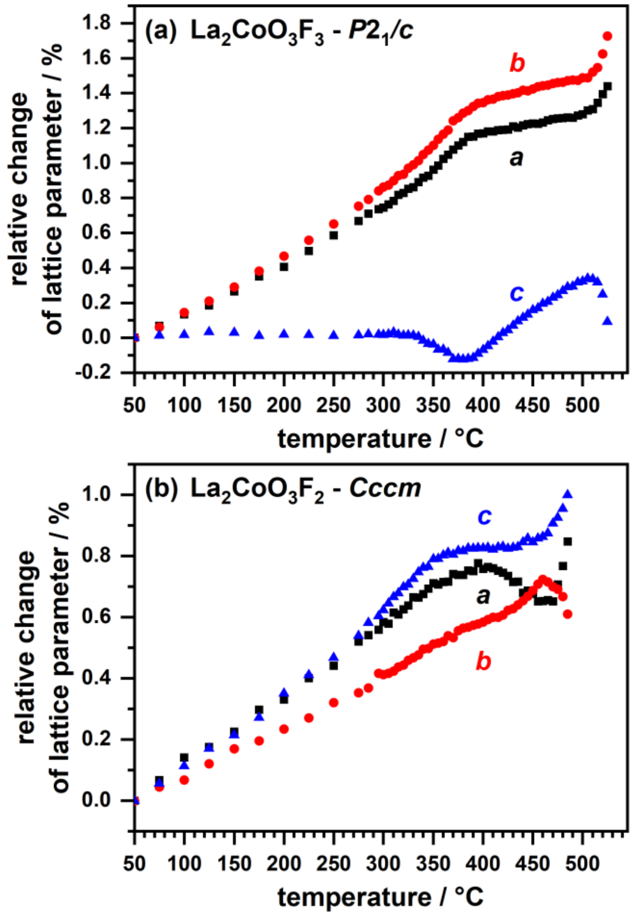

Figure S 13: Relative change of the lattice parameters of $La_2CoO_3F_3$ (a) and $La_2CoO_3F_2$ (b) obtained as function of the temperature. Please note the different coordinate systems of both space groups with the longest axis being *c* in $P2_1/c$ and *a* in *Cccm*.

From the thermal evolution of the lattice parameters extracted from sequential Rietveld refinement (compare Figure S 13) a clear anisotropic thermal unit cell change is apparent for $La_2CoO_3F_3$. For this compound thermal expansion almost exclusively happens in the *a/b* plane (~1.5% increase between 50 °C, and 450°C) while for the longest axis *c* almost no change is observed up to 340 °C. Above this temperature the relative lattice change becomes slightly negative up to 380 °C from where a monotonous increase is obtained and a total relative increase of ~0.3 % is found for *c* between 50 °C, and 450°C. The non-monotonous behavior in *c* might be attributed to changes in the interstitial anion lattice due to increase anion mobility similar to what has been observed for the oxyfluorides of the $La_2Ni_{1-x}Cu_xO_3F_2$ substitution series.[9] For $La_2CoO_3F_2$ an anisotropic thermal unit cell expansion is also observed, here thermal expansion in *a*, and *c* is significantly stronger than the increase in *b* (please note that *a* is the longest axis in *Cccm*). This translates to a decrease

of the orthorhombic unit cell distortion and thus less tilted octahedra during thermal expansion similar to what was observed for $La_2NiO_3F_2$ before[11].

Magnetic characterization of $La_2CoO_3F_3$ and $La_2CoO_3F_2$

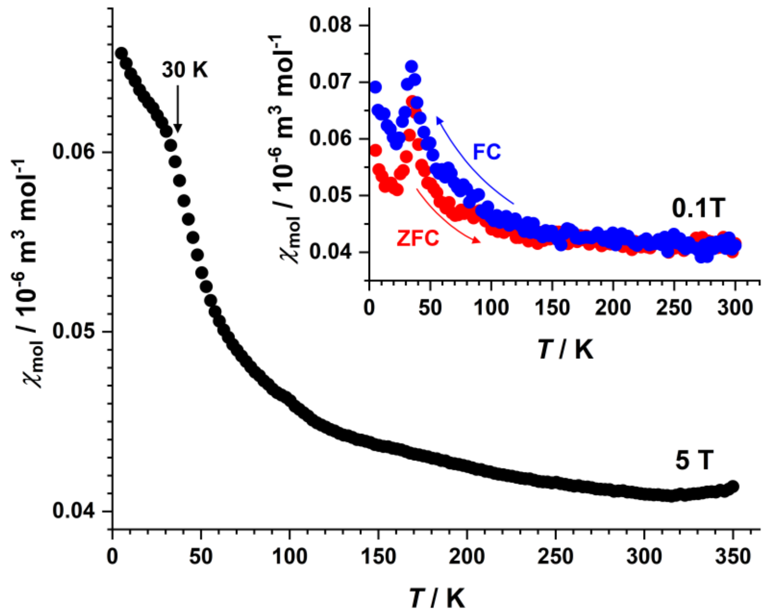

Figure S 14: Susceptibility vs temperature data obtained for La_2CoO_4 in two different external magnetic fields B = 0.1 T, and 5 T. The 0.1 T, that is shown in the inset was obtained inf zero field cooled (ZFC) and field cooled (FC) conditions.

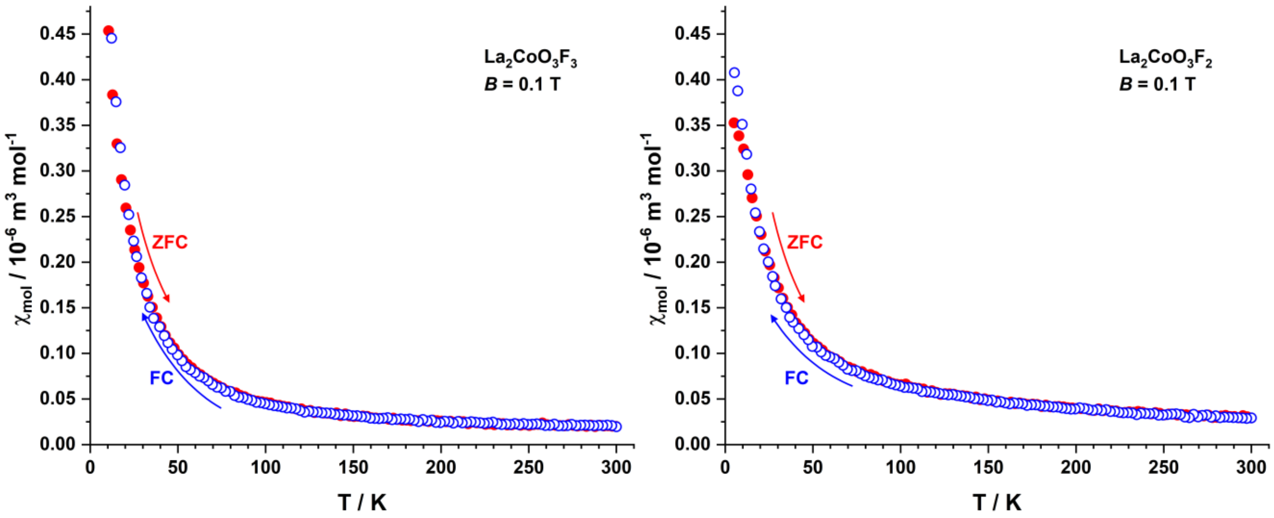

Figure S 15: Susceptibility vs temperature data obtained for $La_2CoO_3F_3$, and $La_2CoO_3F_2$ in an external magnetic field of B = 0.1 T in zero field cooled (ZFC) and field cooled (FC) conditions.

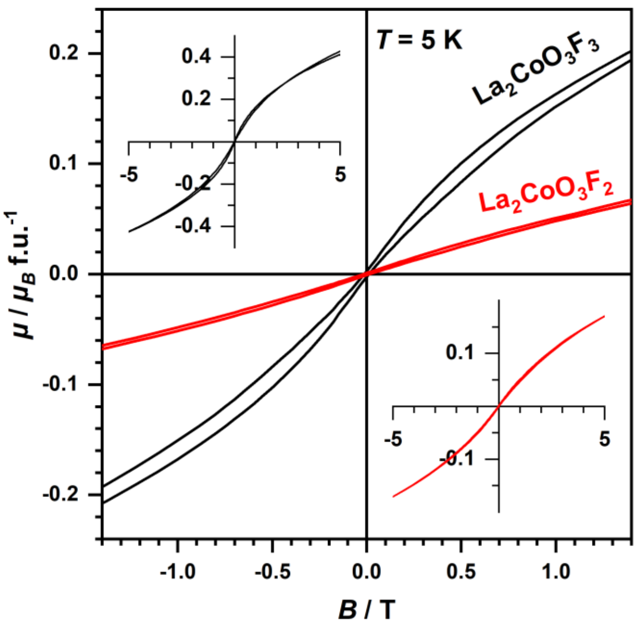

Figure S 16: Magnetic moment vs. field data obtained at 5 K for $La_2CoO_3F_3$, and $La_2CoO_3F_2$.

Refrences

(1) De Barros, R. et al. Reaction Intermediates and Oxygen Ordering Explored by In Situ Neutron Powder Diffraction during Thermal Oxidation of La 2 CoO 4.00. *Inorg. Chem.* **2026**, *65* (2), 1142–1150. doi:10.1021/acs.inorgchem.5c04240.

(2) Jacobs, J. et al. Unveiling the Fluorination Pathway of Ruddlesden-Popper Oxyfluorides: A Comprehensive in Situ X-Ray and Neutron Diffraction Study. *J. Am. Chem. Soc.* **2025**. doi:10.1021/jacs.4c18187.

(3) Wissel, K. et al. Topochemical Reduction of $La_2NiO_3F_2$: The First Ni-Based Ruddlesden–Popper n = 1 T′-Type Structure and the Impact of Reduction on Magnetic Ordering. *Chem. Mater.* **2020**, *32* (7), 3160–3179. doi:10.1021/acs.chemmater.0c00193.

(4) He, Y. et al. Atomic Insights into Topochemical Fluorination and Strong Octahedral Tilt in La 2 CoO 4. *Chinese Phys. B* **2025**. doi:10.1088/1674-1056/adda0a.

(5) Nowroozi, M. A. et al. LaSrMnO4: Reversible Electrochemical Intercalation of Fluoride Ions in the Context of Fluoride Ion Batteries. *Chem. Mater.* **2017**, *29* (8), 3441–3453. doi:10.1021/acs.chemmater.6b05075.

(6) Aikens, L. D. et al. Staged Fluorine Insertion into Manganese Oxides with Ruddlesden-Popper Structures: LaSrMnO4F and La1.2Sr1.8Mn2O7F. *J. Mater. Chem.* **2002**, *12* (2), 264–267. doi:10.1039/b105550j.

(7) Mezzadra, G. et al. The Role of Oxygen Excess on Fluoride Intercalation in Ruddlesden–Popper Electrodes for Fluoride Ion Batteries: The Case of LaSrMnO 4. *J. Mater. Chem. A* **2026**, No. January. doi:10.1039/D6TA00453A.

(8) Nowroozi, M. A. et al. La_2CoO_4: A New Intercalation Based Cathode Material for Fluoride Ion Batteries with Improved Cycling Stability. *J. Mater. Chem. A* **2018**, *6* (11), 4658–4669. doi:10.1039/c7ta09427b.

(9) Jacobs, J. et al. Ruddlesden-Popper Oxyfluorides $La_2Ni_{1-x}Cu_xO_3F_2$ ($0 \leq x \leq 1$): Impact of the Ni/Cu Ratio on the Thermal Stability and Magnetic Properties. *Inorg. Chem.* **2024**, *2*, 2–9. doi:10.1021/acs.inorgchem.4c01330.

(10) Jacobs, J. et al. Ruddlesden-Popper Oxyfluorides $La_2Ni_{1-x}Cu_xO_3F_2$ ($0 \leq x \leq 1$): Impact of the Ni/Cu Ratio on the Structure. *Inorg. Chem.* **2024**, *63* (13), 6075–6081. doi:10.1021/acs.inorgchem.4c00399.

(11) Wissel, K. et al. Topochemical Fluorination of La2NiO4+d: Unprecedented Ordering of Oxide and Fluoride Ions in $La_2NiO_3F_2$. *Inorg. Chem.* **2018**, *57* (11), 6549–6560. doi:10.1021/acs.inorgchem.8b00661.
